# Cell Signaling-Based Classifier Predicts Response to Induction Therapy in Elderly Patients with Acute Myeloid Leukemia

**DOI:** 10.1371/journal.pone.0118485

**Published:** 2015-04-17

**Authors:** Alessandra Cesano, Cheryl L. Willman, Kenneth J. Kopecky, Urte Gayko, Santosh Putta, Brent Louie, Matt Westfall, Norman Purvis, David C. Spellmeyer, Carol Marimpietri, Aileen C. Cohen, James Hackett, Jing Shi, Michael G. Walker, Zhuoxin Sun, Elisabeth Paietta, Martin S. Tallman, Larry D. Cripe, Susan Atwater, Frederick R. Appelbaum, Jerald P. Radich

**Affiliations:** 1 Nodality, Inc., South San Francisco, California, United States of America; 2 University of New Mexico Cancer Center, Albuquerque, New Mexico, United States of America; 3 SWOG Statistical Center, Fred Hutchinson Cancer Research Center, Seattle, Washington, United States of America; 4 ECOG Coordinating Center, Frontier Science, Boston, Massachusetts, United States of America; 5 Montefiore Medical Center North Division, Bronx, New York, United States of America; 6 Memorial Sloan-Kettering Cancer Center, New York, New York, United States of America; 7 Indiana University Simon Cancer Center, Indianapolis, Indiana, United States of America; 8 Stanford University, Palo Alto, California, United States of America; 9 Fred Hutchinson Cancer Research Center, Seattle, Washington, United States of America; The Ohio State University, UNITED STATES

## Abstract

Single-cell network profiling (SCNP) data generated from multi-parametric flow cytometry analysis of bone marrow (BM) and peripheral blood (PB) samples collected from patients >55 years old with non-M3 AML were used to train and validate a diagnostic classifier (DX_SCNP_) for predicting response to standard induction chemotherapy (complete response [CR] or CR with incomplete hematologic recovery [CRi] versus resistant disease [RD]). SCNP-evaluable patients from four SWOG AML trials were randomized between Training (N = 74 patients with CR, CRi or RD; BM set = 43; PB set = 57) and Validation Analysis Sets (N = 71; BM set = 42, PB set = 53). Cell survival, differentiation, and apoptosis pathway signaling were used as potential inputs for DX_SCNP_. Five DX_SCNP_ classifiers were developed on the SWOG Training set and tested for prediction accuracy in an independent BM verification sample set (N = 24) from ECOG AML trials to select the final classifier, which was a significant predictor of CR/CRi (area under the receiver operating characteristic curve AUROC = 0.76, p = 0.01). The selected classifier was then validated in the SWOG BM Validation Set (AUROC = 0.72, p = 0.02). Importantly, a classifier developed using only clinical and molecular inputs from the same sample set (DX_CLINICAL2_) lacked prediction accuracy: AUROC = 0.61 (p = 0.18) in the BM Verification Set and 0.53 (p = 0.38) in the BM Validation Set. Notably, the DX_SCNP_ classifier was still significant in predicting response in the BM Validation Analysis Set after controlling for DX_CLINICAL2_ (p = 0.03), showing that DX_SCNP_ provides information that is independent from that provided by currently used prognostic markers. Taken together, these data show that the proteomic classifier may provide prognostic information relevant to treatment planning beyond genetic mutations and traditional prognostic factors in elderly AML.

## Introduction

In “elderly” AML populations (typically defined by age >55 or >65 years), the complete remission (CR) rate in response to standard-dose cytarabine (Ara-C)-based induction chemotherapy ranges from 35 to 50% depending on the study, while the rate of treatment-related mortality (TRM) ranges from 15–20% [1], [2]. Other induction-therapy options for elderly AML patients, including high-dose Ara-C [3], high-dose daunorubicin [4], hypomethylating agents or other investigational agents have been shown to increase the rate of CR to 40–50% [4]. The ability to distinguish patients likely to benefit from standard induction therapy from those likely to fail such therapy would be a significant contribution to patient management, by allowing patients to avoid harmful treatment that is likely to be futile, perhaps in favor of enrollment in clinical trials evaluating new targeted and less intensive regimens as first line treatment. Considerable effort has gone into creating models based on clinical parameters, cytogenetics and molecular testing to predict response [2], [5]. Technologies such as FISH and rapid molecular testing aim at making established diagnostic methods (such as cytogenetics and detection of leukemogenic mutations) which can assist in the risk classification and prognostication of AML available to patients earlier in the diagnostic process. However, in community practice and non-academic treatment centers where a considerable proportion of elderly AML patients are treated, cytogenetic and molecular test results are not always available at the time of the initiation of induction therapy [6]due to a longer turn-around time between sample acquisition and availability of results.

**Fig 1:**
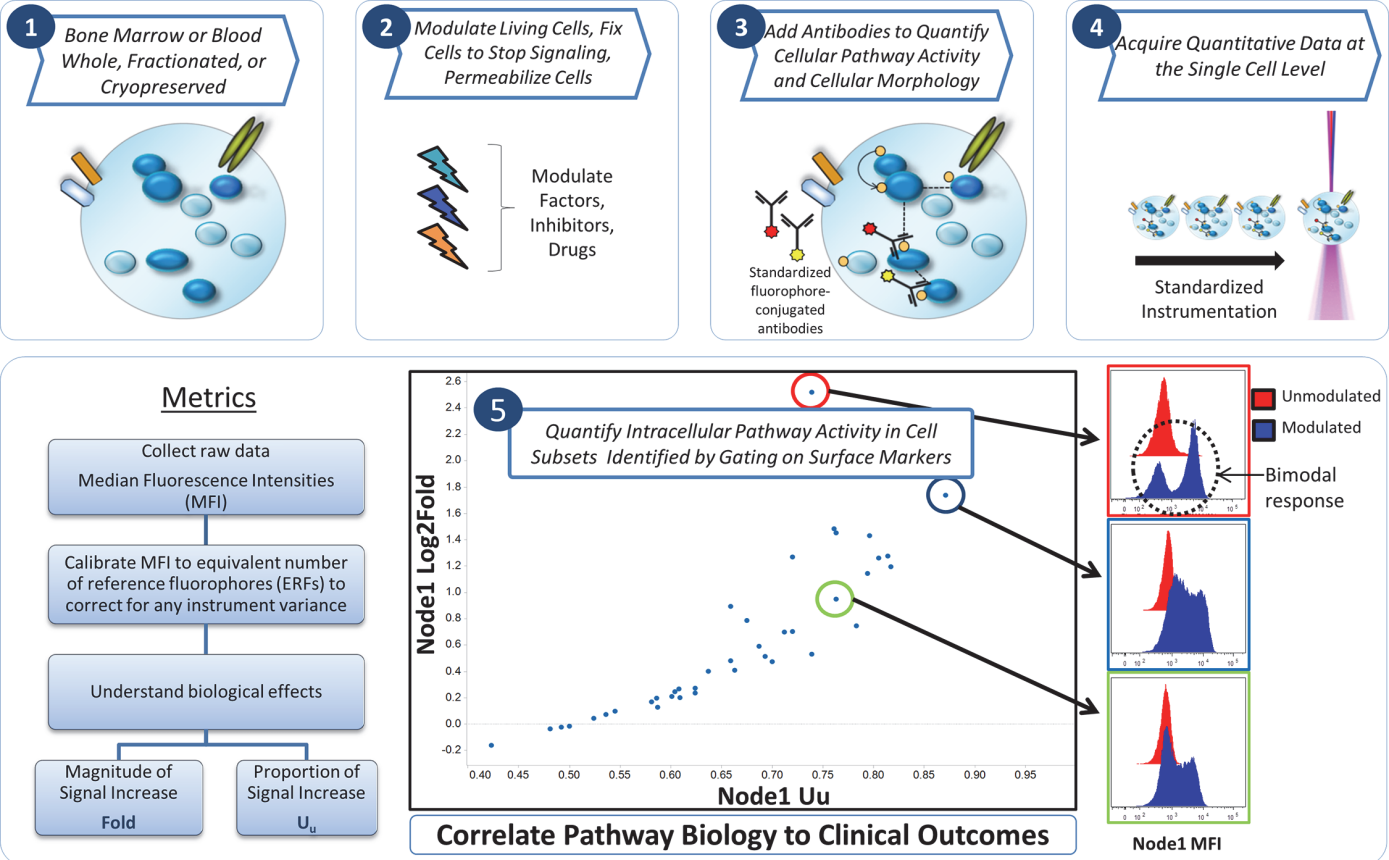
Single Cell Network Profiling (SCNP) Technology. Bone marrow or blood cells (1) are modulated, fixed and permeabilized (2), then stained with an antibody cocktail containing antibodies directed against both cell surface markers as well as post-translational modifications of intra-cellular proteins(3). Cells are acquired using multiparametric flow cytometry (4) thus allowing quantification of intracellular pathway activity in cell subsets identified by gating on lineage surface markers (5). Various metrics to quantify basal and induced signaling and to assess association with biologic and clinical outcomes are applied.

Single-cell network profiling (SCNP) technology uses multi-parameter flow cytometry to study signaling pathways and networks at the single-cell level (Fig 1). Assaying cells at this level of resolution allows the identification of rare cell populations and reveals differences in the capacity of signaling pathways among cell subtypes, as well as between and within patient samples. The feasibility of applying SCNP in various hematologic malignancies has previously been documented [7], [8]. Specifically, an SCNP assay predicting the likelihood of response to induction therapy in pediatric AML has been validated and published [9]. Functional characterization of the disease samples at the single cell level may add information that is independent of existing molecular prognostic factors like *FLT3-ITD* mutation status [10], [11]. The current study presents the development and validation of a SCNP classifier (DX_SCNP_) for the prediction of response to Ara-C-based induction chemotherapy in elderly (> 55 year old) patients with newly diagnosed AML.

## Materials and Methods

### Ethics Statement

In accordance with the Declaration of Helsinki, all patients provided written informed consent for the collection and use of their samples for research purposes. Institutional Review Board approval was obtained from Independent Review Consulting, Inc. (Approval No. 09068–01) on August 31, 2009. Clinical data were de-identified in compliance with Health Insurance Portability and Accountability Act regulations.

### Study Inclusion Criteria and Patient Samples

The study used cryopreserved pretreatment bone marrow (BM) and peripheral blood (PB) samples collected from two groups of AML patients: patients enrolled in SWOG studies (used in the training and validation efforts) and patients enrolled in ECOG studies (used in the verification analysis).

**Fig 2:**
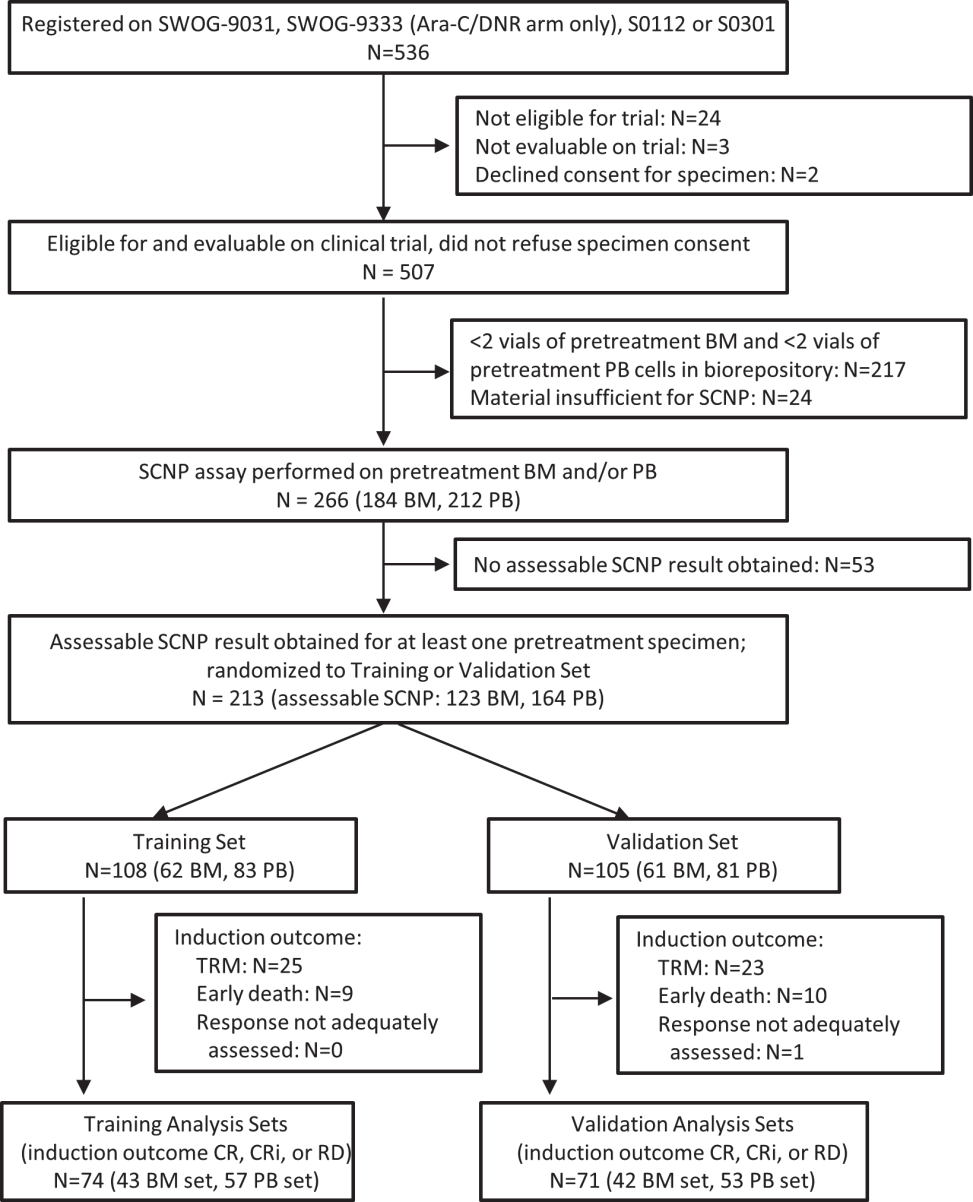
SWOG Patient Disposition. A flow diagram showing all patients enrolled onto the SWOG parent AML trials and rationale for exclusion of patients from the final analysis sets. Text boxes describe the characteristics of patients carried forward.

For patients enrolled in SWOG trials, inclusion criteria were age > 55 years, diagnosis of non-APL AML, and enrollment in one of four SWOG frontline treatment trials using Ara-C-based induction therapy (SWOG-9031 [12], SWOG-9333 [13] (Ara-C/daunorubicin arm only), S0112 [14] or S0301 [14] (S1 Table). Eligible patients had two or more vials of a pre-induction sample (BM, PB or both) remaining in the SWOG AML biorepository, received at least one dose of Ara-C and consented for research use of their samples. Fig 2 shows the SWOG patient disposition flowchart: of the 536 patients registered on the above mentioned SWOG trials, 266 patients contributed samples (i.e. pretreatment BM and/or PB) to the SCNP assay.

**Fig 3:**
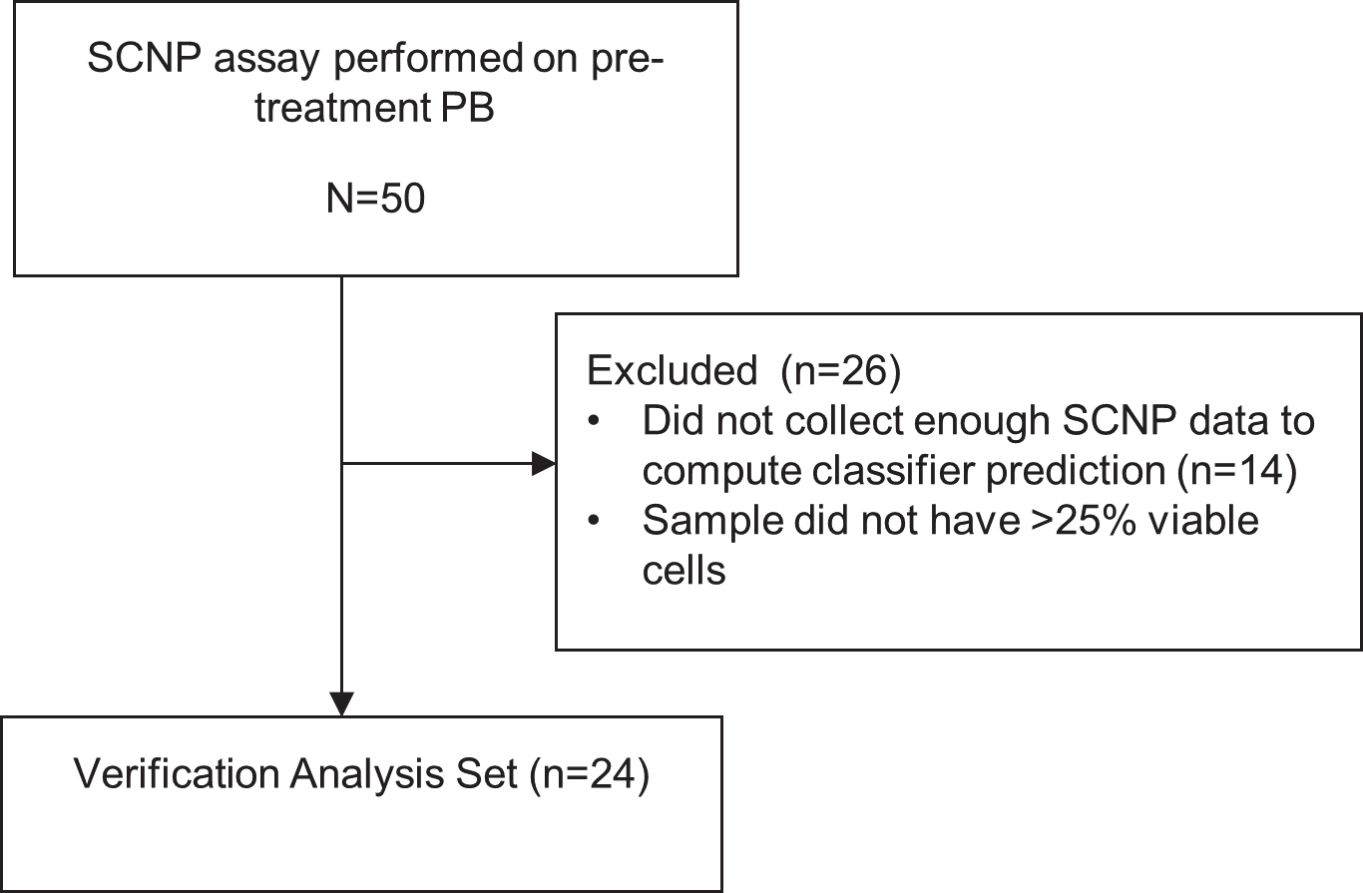
ECOG Patient Disposition. A flow diagram showing all patients enrolled onto the parent ECOG AML trials and rationale for exclusion of patients from the final analysis sets. Text boxes describe the characteristics of patients carried forward.

For patients enrolled in ECOG trials, inclusion criteria were > 60 years of age with diagnosis of non-APL AML enrolled in one of two ECOG treatment protocols using Ara-C-based induction therapy: E3993 [15] and E3999 [16] (S1 Table). Eligible patients had two or more remaining vials of a pre-induction BM sample stored in the ECOG AML tissue repository, received at least one dose of Ara-C, and consented for research use of their samples. Fig 3 shows the ECOG patient disposition flowchart: 50 patients contributed a BM sample to the Verification Set.

Induction therapy for all patients consisted of a variation on standard dose cytarabine-based therapy (100-200mg/m^2^) for 7 days and daunorubicin 30-45mg/m^2^ for 3 days (for details of study designs including sample size, chemotherapies received and response see S1 Table).

For all studies, the following induction-therapy outcomes were defined, based on previously published guidelines [6]: complete response (CR); CR with incomplete peripheral blood count recovery (CRi); resistant disease (RD); and TRM, including fatal induction toxicity, and early death (ED) in the absence of fatal induction toxicity (i.e., treatment response coded as death from any cause by study day 30 or indeterminate due to death during aplasia or within 7 days after induction). The CR rates for the treatment regimens from SWOG and ECOG studies referenced above ranged from 38% to 50% [12], [13], [14], [15], [16].

Cytogenetic risk classification was first determined per the SWOG study from which the samples were obtained for sample randomization (stratification purposes). Cytogenetic risk classification for all samples (SWOG and ECOG) were then assigned using NCCN 2013 guideline criteria for purposes other than randomization (e.g. model building DX_CLINICAL2_). Similarly to what is done in clinical practice, patients with unknown cytogenetics risk categories were imputed as intermediate cytogenetics.

### Study Design

**Fig 4:**
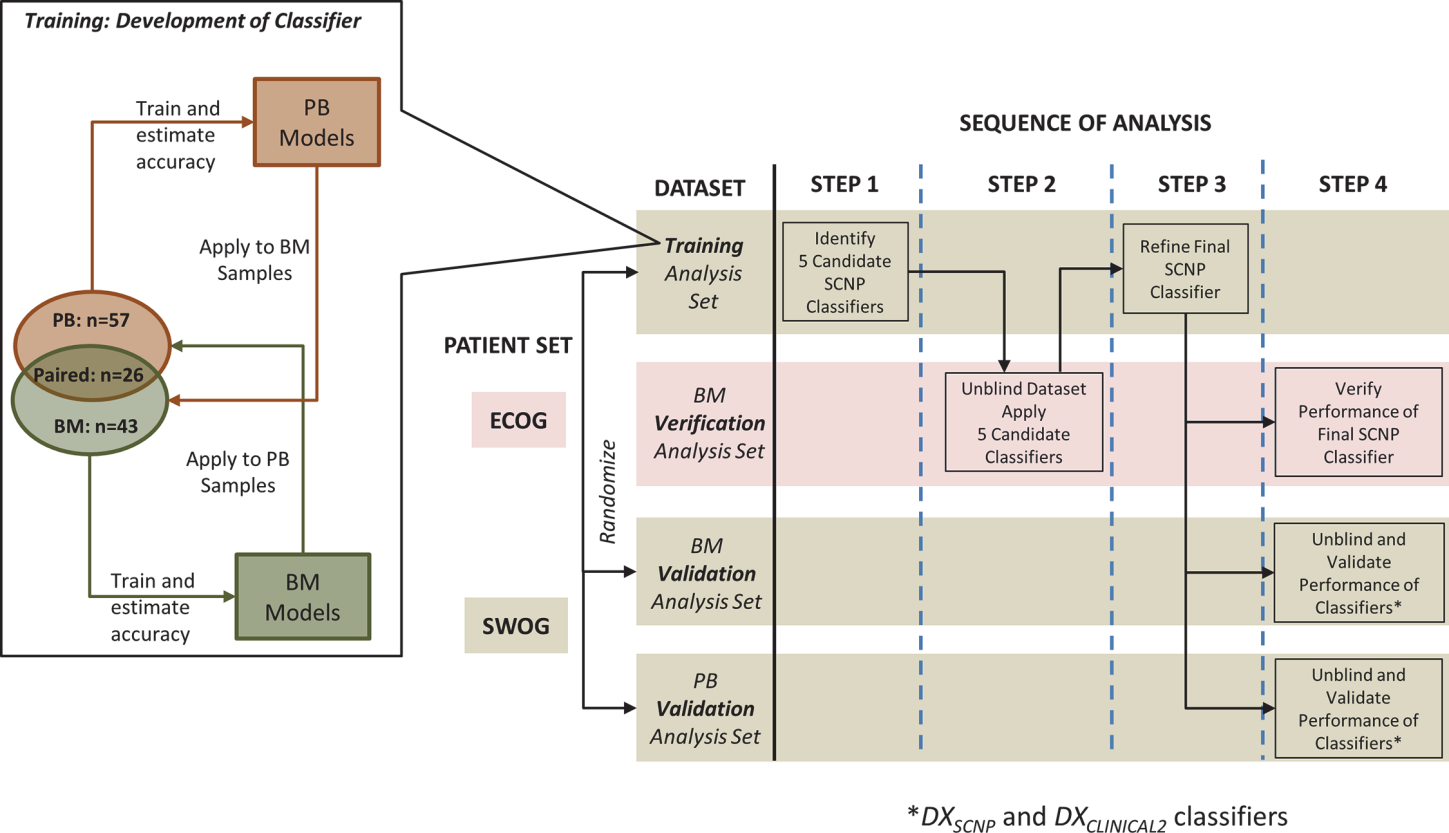
Study Design Diagram. Flowchart of the study design with descriptive schematics of the patient sets in the Training, Verification and Validation analysis sets. SWOG samples were randomized into a Training and a Validation Analysis set and were sorted by tissue type (PB or BM). An initial subset of classifiers was trained separately in PB and BM samples in the Training Analysis sets and then PB classifiers were applied to BM and BM classifiers were applied to PB. From this training process 5 candidate classifiers were selected and applied to the ECOG Verification Analysis set. The final SCNP classifier was further refined and applied to 1) ECOG Verification Analysis set, 2) SWOG BM Validation Analysis set and 3) SWOG PB Validation Analysis set.

SCNP assays for all SWOG patient samples were performed blindly to all clinical data and in a random order as part of a single experiment. Patients with assessable BM and/or PB SCNP results (n = 213) were then randomized approximately 1:1 to Training and Validation Sets (see Figs 2 and 4). The minimization approach of Pocock and Simon [17] was used to balance disease characteristics and other relevant variables between the Training and Validation Sets. These included: response to induction therapy, sample type(s), cytogenetic risk group assigned per SWOG protocol, SWOG parent trial treatment arm, and FLT3-ITD mutation in BM and/or PB samples, and extent of proteomic readout availability in BM and/or PB samples (see S1 Methods). Within the Training and Validation Sets, only patients having an induction outcome of CR, CRi, or RD were assigned to the Training and Validation Analysis Sets; patients with TRM were excluded from the Analysis Sets since the assay was specifically designed to measure blast chemosensitivity and not comorbidities [9], [18] (Fig 2). Clinical and molecular variables from 74 patients randomized to the Training Set were used to develop DX_CLINICAL1_ and DX_CLINICAL2_. SCNP data for the BM (n = 43) and PB (n = 57) Training Analysis Sets were used to develop the SCNP-based predictive models. Since some patients had two SCNP-assessable samples (BM and PB), a partial overlap existed between the patients in the BM and PB Analysis Sets. However, within each Analysis Set, each patient contributed only one sample (i.e., only one tissue type) (Fig 4). Values of inputs for DX_CLINICAL1_ and DX_CLINICAL2_ which were missing (≤6% for any input except for cytogenetics which was missing in approximately 20%), were imputed as described in S3 Methods.

Patient BM samples from ECOG AML trials were assayed separately and were used as an independent BM Verification Set to test multiple candidate classifiers prior to locking the final DX_SCNP_ classifier for validation (Figs 3 and 4).

### SCNP Assay Terminology and Biological Pathways Evaluated in Training Set

For detailed information on assay components and performance parameters please see S1 MiFlowCyt Report compiled as per MiFlowCyt guidelines [19].

**Fig 5:**
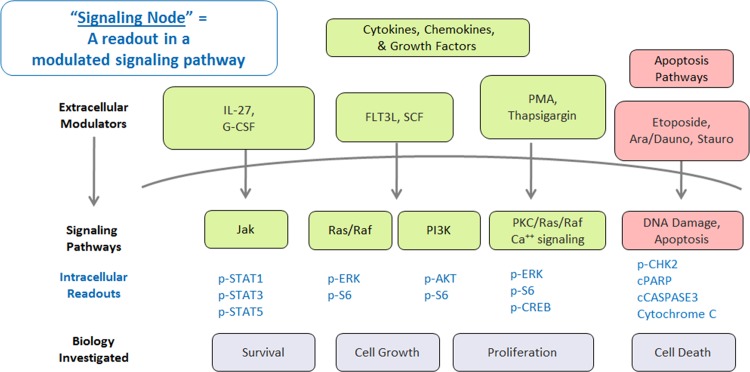
Pathways investigated using SCNP in the Training study. Schematic of the cell signaling pathways probed in the Training set. An SCNP node consists of the combination of a modulator and the corresponding intracellular readout. Modulators are shown outside the cell initiating signaling pathways that produce an intracellular proteomic response (readouts shown below the curve indicating cell membrane).

Based upon relevance to AML pathophysiology, cell surface receptors and signaling pathways involved in cell survival, proliferation, programmed cell death (apoptosis), and the DNA damage response (DDR) were investigated. Apoptotic signaling and DDR pathways were measured after *in vitro* exposure of AML samples to etoposide or Ara-C (Fig 5).

The term “signaling node” (or simply “node”) refers to a proteomic readout in the presence or absence of a specific modulator at a specific time point after modulation. Modulators included endogenous growth factors (e.g., FLT3 ligand), cytokines (e.g., IL-27) and drugs (cytarabine, daunorubicin, and etoposide). Several metrics (normalized assay readouts, see metrics section and S1 Fig) were applied to quantify the amplitude of response of each signaling node.

### SCNP Assay

**Fig 6:**
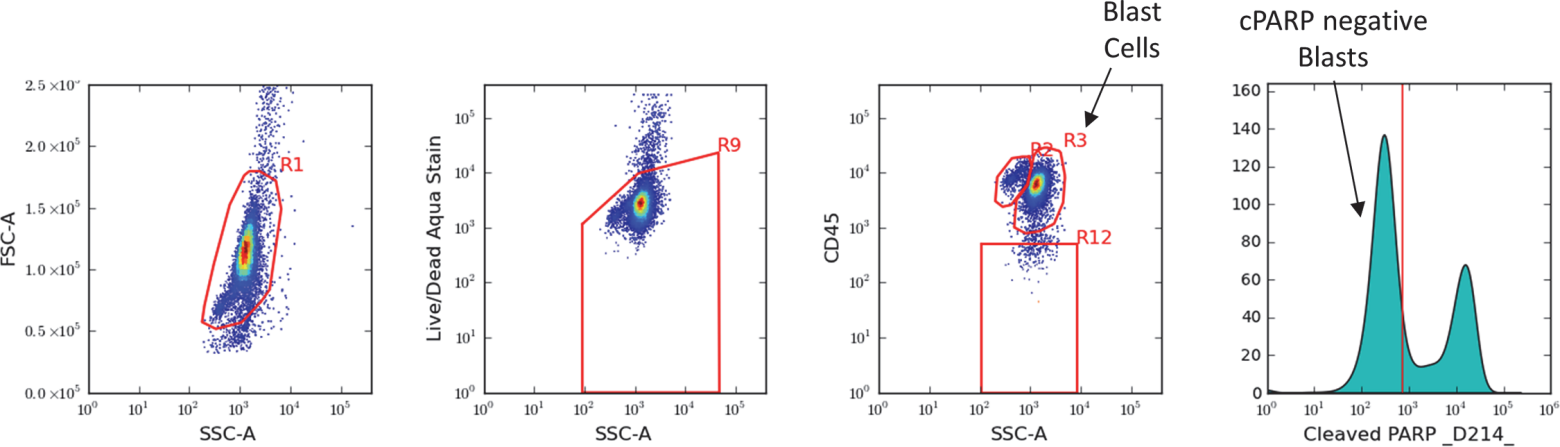
Gating: Identification of Blast Cell Population. Illustration of gating to identify blast cell population and cPARP negative blast cells. Intact cells were identified using scatter. Amine Aqua was then used to identify viable cells. CD45 was then used to identify Blast Cells. In wells where short term signaling was assayed, the blast cells were gated using cPARP expression to identify healthy leukemic cells.

The assay was conducted over a 9 week period with 2 batches of 28 samples tested per week. Cells were incubated in 96-well plates according to a pre-specified node priority to evaluate a total of 9 modulators and 53 signaling nodes (see S1 Inputs and S1 MiFlowCyt Report) with 100,000 cells per well. Cells were fixed, permeabilized, and incubated with a cocktail of fluorochrome-conjugated antibodies that recognize extracellular lineage markers and intracellular epitopes. To assess cell maturation and viability, anti-CD34, anti-CD45 antibodies and Amine Aqua (AA) stain were included in each well. To assess cell “health” [10], anti-cleaved-PARP antibody (cPARP) was included in every well to allow gating (Fig 6) on cPARP negative (i.e., non-apoptotic) leukemic blast cells. An example of one SCNP assay “node” is: AML cells were incubated with FLT3 ligand (modulator) for 15 minutes and after fixation and permeabilization were exposed to a cocktail of antibodies against surface linear markers (CD45 and CD34) and against epitope-specific sites for the following proteins: cPARP, p-AKT, p-ERK, p-S6.

After completion of the SCNP assay, pre-specified methods were used to determine sample evaluability. The term “SCNP-assessable” refers to samples meeting pre-specified assay inclusion and evaluability criteria. SWOG patients with no SCNP-assessable sample(s) were designated non-evaluable and excluded from analyses (see Fig 2). Clinical characteristics of the 213 SCNP-evaluable SWOG patients and the 294 SWOG patients who were ineligible for this study (N = 241) are compared in S2 Table. Statistically significant differences in some clinical characteristics including WBC and percent of leukemic blasts in both BM and PB (all higher in the evaluable subset, reflecting the greater availability of repository samples from patients with higher counts) were observed.

### Data and Software

Data were acquired using FACSDiva software (BD Biosciences, San Jose, CA) on several Canto II (BD) FACS Canto II flow cytometers. FCS files were gated using WinList (Verity House Software, Topsham, ME) and all data were stored in a MySQL database for access and querying. For details please refer to S1 MiFlowCyt Report.

### Metrics

Specific metrics were developed to describe and quantify the functional changes observed using the SCNP assay. Median fluorescence intensity (MFI) was computed from the fluorescence intensity levels of the cells. Equivalent Number of Reference Fluorophores (ERF), a transformed value of the MFI values, was computed using a calibration line determined by fitting observations of a standardized set of 8-peak rainbow calibration particle beads (RCPs) for all fluorescent channels (Spherotech Libertyville, IL; Cat. No. RFP-30-5A) to standard values assigned by the manufacturer. ERF was used to standardize, qualify and monitor the instrument during setup, and to calibrate the raw fluorescence intensity readouts on a plate-by-plate basis and to control for instrument variability. ERF values were then used to compute a variety of metrics to measure the biology of functional signaling proteins (S1 Fig). Additional metrics to measure total phospho-protein levels before and after modulation were computed using ERF value between two wells as listed below. In the metric definitions that follow a = autofluorescence, u = unmodulated, and m = modulated.

- Basal is defined as:

$$\text{Basal}=\log_{2}\left[\frac{ERF_{\text{unmodulated}}}{ERF_{\text{autofluorescence}}}\right]$$

- Log2Fold Change is defined as:

$$\log2\text{Fold}=\log_{2}\left[\frac{ERF_{\text{modulated}}}{ERF_{\text{unmodulated}}}\right]$$

- U_u_ is the Mann-Whitney U statistic comparing the intensity values for an antibody in the modulated and unmodulated wells that has been scaled to the unit interval (0,1) for a given cell population for a sample. Measures the proportion of cells that have higher (U_u_ > 0.5) or lower (U_u_ < 0.5) expression of the antibody in the modulated state compared to the unmodulated state. Similar in nature to percentage of cell that have positive (or negative) expression, except that a threshold to determine positivity is not necessary.
- U_a_ is the same as the U_**u**_ metric except that the auto-fluorescence well is used as the reference instead of the unmodulated well.

The percent healthy metric was calculated for each sample by using the autofluorescence well and the c-PARP stained well:

$$P_{h}^{Intact}:$$

Percentage of leukemic blast cells that is negative for cPARP expression. The 98^th^ percentile value for autofluorescence was used to determine the positive-negative split point.

### Controls and Reproducibility

Standard instrument controls (RCP beads—see section above and S1 MiFlowCyt Report) and cell line controls (see below) enabled the assessment of technical variability at the modulation, fixation, staining, and acquisition steps in the laboratory work flow thus allowing for the generation of reproducible results across operators, plates and time. These controls are essential in clinically applicable assays. Overall assay performance was monitored by running GDM1 and RS4; 11 cell lines on every plate. Original cell lines were obtained from American Type Culture Collection (ATCC; Manassas, VA). A single batch of these cell lines were expanded in culture, cryopreserved, quality control tested and released following performance verification according to approved SOPs and appropriate release specifications. Intra- and inter-cytometer variance and longitudinal consistency of instrument performance were monitored by including a single lot of 8-peak RCPs on each plate across the entire experiment. Additionally, all cytometers were qualified each day before use according to the manufacturer’s suggested quality control program as well as a more stringent internally developed quality control program documented in approved SOPs and performance specifications. Cytometers performing outside Nodality’s established performance specifications were taken off-line, corrective actions taken and documented and the instrument then verified prior to bringing back on-line for use. With these controls in place the majority (28/44) of the CVs were less than 5% and most of them (42/44) were less than 10% as expected across all days and batches for the study (S1 MiFlowCyt Report).

### Classifier Development

During the training phase, clinical data for the Training Set were unblinded and used to develop three distinct classifiers, DX_CLINICAL1_, DX_CLINICAL2_, and DX_SCNP_, using different sets of inputs to predict CR/CRi vs. RD.

#### 1. DX_CLINICAL1_

Inputs included clinical factors available at diagnosis [i.e. age, BM blast percentage, white blood cell count, peripheral blast percentage, neutrophil and monocyte counts (percent and absolute), hemoglobin, platelet count, performance status (0–1 vs. 2–3), FAB class (M0/M1/M2/M7 vs. other), and AML onset (de novo vs. secondary)] for the 74 patients from SWOG trials randomized to the Training Set.

#### 2. DX_CLINICAL2_

In addition to the clinical factors used for DX_CLINICAL1_, inputs for DX_CLINICAL2_ included cytogenetic risk group, percentage of CD34+ cells, and presence of genetic markers for FLT3-ITD (as a continuous variable and as binary) and NPM1 mutations. Percentage of CD34+ cells was calculated from phenotyping well during the SCNP assay because this information was not available for the SWOG samples from the clinical site.

#### 3. DX_SCNP_

Inputs included cell survival, growth, differentiation, and cell-death pathways with multiple modulated proteomic readouts (e.g., IL-27→p-STAT1/3/5; Ara-C/daunorubicin→cPARP for a total of 53 signaling nodes (Fig 5). Since only a subset of the overall available samples were “paired” (i.e. BM and PB samples collected from the same patient) and based on our previous published data showing that the correlation of signaling nodes in paired PB and BM samples, although high, was not perfect in particular in AML secondary to MDS [20], the decision was made to perform DX_SCNP_ modeling separately in the BM (n = 43) and PB (n = 57) Training Analysis Sets and then to assess, by cross applying the classifiers from one tissue type to the other in the training set, if independent classifiers were needed for each tissue type. Logistic regression models were investigated as predictors of response. These models were fit by LASSO using the R Penalized Package [21], and their performance was measured by the AUROC, using out-of-bag (OOB) estimation. Model outputs for all classifiers were continuous scores, where higher scores indicated a greater probability of response (CR/CRi) to Ara-C-based induction chemotherapy.

Fig 4 summarizes the workflow followed from model training to validation for DX_SCNP_. Briefly, an initial subset of nodes was first identified in each Training Set by examining: 1) node signaling differences between CR/CRi and RD, 2) Random Forest [22] node importance for CR/CRi vs. RD using all nodes, and 3) non-zero node coefficients by Penalized Logistic Regression [23], [24], [25], [26], [27] for CR/CRi vs. RD using all nodes.

To find combinations of nodes that were better predictors of CR/CRi vs. RD compared to individual nodes, models based on 2-to-4 node combinations from the initial node subset were developed using logistic regression. The adjusted AUROC for each of the models was calculated [28]. Bootstrap re-sampling (n = 500) was used to adjust the AUROC for optimism. The models were then ranked by their adjusted AUROC and several related high-ranking models were investigated further. The lead candidates were selected based on several criteria, including biological pathway relevance, range of node signaling and experience from previous studies [7].

Five candidate DX_SCNP_ classifiers (Table 1), each containing apoptosis pathway nodes alone or in combination with signaling nodes, were generated using the PB Training Analysis Set. The AUROCs for these models, when applied to the BM Training Analysis Set, were all greater than 0.74, justifying their use for both tissue types. The five candidate models were then locked for evaluation in the Verification Analysis Set (BM samples from ECOG trials), and the resulting data were used to select a single DX_SCNP_ classifier to refine and lock for validation (Table 1). The out-of-bag (OOB) estimates of AUROC for this final classifier were 0.81 and 0.89 in the BM and PB samples respectively. Among donors with paired PB and BM samples, the AUROC values were 0.86 and 0.93 in BM and PB respectively.

**Table 1:** Candidate Models.

Model Description				AUROC (Out of Bag) in Training Analysis Sets		AUROC in BM Verification Analysis Set
Model	Component 1	Component 2	Component 3	PB	BM	BM^a^
1	AraC+D→cPARP | U_u_	FLT3L→p-Akt | log_2_(Fold)	-	0.91	0.78	0.67 (p = 0.119)
2	AraC+D→cPARP | U_u_	PMA→pCREB | log_2_(Fold)	-	0.82	0.75	0.65 (p = 0.153)
3	AraC+D→cPARP | U_u_	AraC→CD34 | U_u_	FLT3L→p-Akt | log_2_(Fold)	0.91	0.81	0.72 (p = 0.047)
4	AraC+D→cPARP | U_u_	FLT3L→p-Akt | log_2_(Fold)	Basal→p-Akt | U_a_	0.86	0.74	0.67 (p = 0.084)
5	AraC+D→cPARP | U_u_	AraC→CD34 | U_u_	-	0.91	0.8	0.76 (p = 0.017)

a) The number of patients who achieved CR/CRi varied between 8 and 12 for each of the classifiers depending on the availability of node-metric data of all the predictor variables involved in a classifier. The number of RDs was 12 for all models.

The final selected Elderly AML Induction Response classifier was locked (Table 2) and classifier scores were calculated for each patient in the BM Validation Analysis Set (n = 42) and PB Validation Analysis Set (n = 53) independently. Prior to finalizing the validation analysis plan, the option to combine the tissue types to increase the sample size was considered but not chosen to avoid an arbitrary choice of tissue type for the donors with both PB and BM samples. For the donors with both PB and BM samples, the predictions from DX_SCNP_ were compared visually by generating a scatter plot and computing Pearson correlation coefficient.

**Table 2:** Locked DX_SCNP_ Classifier Inputs.

Component	Coefficient	Relationship	Node-metric	Modulator	Time	Antibody	Metric
C_1_	95.60133	C_1_ = (N_1_–0.5)^2^ **if** N_1_ > 0.5 **else** C_1_ = 0.0	N_1_	AraC+Dauno	24 Hours	cPARP	U_u_
C_2_	34.94358	C_2_ = (0.5—N_2_)^2^ **if** N_2_ <0.5 **else** C_2_ = 0.0	N_2_	AraC+Dauno	24 Hours	CD34	U_u_
Intercept	-1.26004	-	-	-	-	-	-

*Score =* $\frac{e^{\chi^{'}\hat{\beta }}}{1+e^{\chi^{'}\hat{\beta }}}$ *where* $\chi^{'}$ *is the vector of node-metric values and* $\hat{\beta }$ *is the vector of regression coefficients*

A similar procedure was followed to build DX_CLINICAL1_ and DX_CLINICAL2_. However, since a majority of inputs for these predictors are not tissue-specific, separate models for BM and PB were not considered. Data from all patients in the Training Analysis Set (N = 74) were input to penalized regression methods to identify variables that are likely to be predictive of response. In the case for DX_CLINICAL1_, none of the input variables were chosen by penalized regression (i.e. all coefficients were shrunk to zero), indicating that a classifier cannot be constructed with these input variables. For DX_CLINICAL2_, performance characteristics were estimated using bootstrapping in the Training Analysis Set in the same manner as done for DX_SCNP_. DX_CLINICAL2_ was applied independently to the BM and PB Validation Analysis Sets, as was done for DX_SCNP_.

### Statistical Analysis

Patient and disease characteristics were summarized by standard descriptive techniques, and compared between subsets using Fisher’s exact test, Pearson’s chi-squared test of independence, logrank, and the Mann-Whitney test.

### Sample Size

Estimates of AUROC, after adjusting for optimism, from the Training Analysis Set for the lead DX_SCNP_ candidates were between 0.74 and 0.91 (Table 1). Sample size estimates for the test of AUROC against the value of 0.5 under null hypothesis were performed for true AUROC in the range of 0.75 and 0.80. The power was expected to exceed 80% for a Validation Analysis Set of approximately 50 subjects with the same induction response rate as the subjects in the Training Analysis Set.

### Measures of Predictive Performance

Since the AUROC, estimated empirically using the trapezoidal method, is equivalent to the Mann-Whitney U-statistic (AUROC = U/(n_1_+n_2_) [27]), the null hypothesis of no association (H0: U = 0) tested against the one sided alternative (H1: U>0), is equivalent to a one-sided exact test of H0: AUROC = 0.5 versus H1: AUROC>0.5. In addition to the p-value from the exact test for the Mann Whitney U value, the 95% confidence interval for the AUROC estimate was calculated using the bias-corrected-and-accelerated (BCa) bootstrap method [29].

### Response Prediction using Cell Death as Measured by Amine Aqua

Amine aqua stain was included in every well to allow exclusion by gating of non-viable cells. To assess whether a measurement of cell viability alone after 24 hour incubation with AraC/daunorubicin could accurately predict induction outcome, a decrease in the viability of cells treated with Ara-C/Daunorubicin for 24 hours relative to untreated cells (at 24 hours but otherwise processed similarly) was used for this purpose. The U_u_ metric was used to measure this change in viability and then used to assess an association with response to therapy.

## Results

### Patient Characteristics

As shown in Fig 2, assessable SCNP results were obtained for at least one specimen for 213 SWOG patients, and for 24 patients in the ECOG Verification Set (Fig 3). Characteristics of patients in the BM Training, Verification and Validation Analysis Sets and the of the PB Training and Validation Analysis Sets are shown in Tables 3 and 4, respectively. No statistically significant differences in clinical characteristics were observed between the Training and Validation Sets, or between SCNP-evaluable and-nonevaluable patients (Table 5 and 6 for SWOG and ECOG patients, respectively). Of note, although not statistically significant, the BM Training Analysis Set had a lower percentage of patients with secondary AML (12%) compared with the BM Validation Analysis Set (19%) (secondary AML was not a stratification factor in the sample randomization) and the BM Verification Analysis Set (29%). By contrast, comparison between the 213 SCNP-evaluable SWOG patients and the 294 other potential SWOG patients (53 selected for the study but with no SCNP-assessable results, 241 not selected primarily due to fewer than 2 vials available from the repository) showed that SCNP-evaluable patients had significantly higher counts (WBC, BM and PB blast percentages), fewer patients from SWOG-9031 (earliest of the four SWOG trials), and fewer patients with monosomy 5 or 7 (S2 Table). However, treatment outcomes did not differ significantly between the two groups.

**Table 3:** Patient/Disease Characteristics of the BM Training, Verification and Validation Analysis Sets.

Patient/Disease Characteristics	Subgroups	BM Training Analysis Set (n = 43)	BM Validation Analysis Set (n = 42)	P^b^	BM Verification Analysis Set (n = 24)
**Response to induction therapy** ^**a**^	RD	12	10	0.92	12
	CR/CRi without CCR1	19	20		12
	CCR1	12	12		0
**Age (Years)**	(Min, Max), Median	(56.8, 83.9), 68.2	(58.0, 82.0), 69.0	0.98	(57, 80), 69
**Cytogenetic risk group**	Better	4	2	0.86	0
	Intermediate^c^	26	25		10
	Poor	6	8		5
	Missing	7	7		9
**FLT3 ITD**	Mutant	8	10	0.60	4
	Wildtype	35	32		17
	Unknown	0	0		3
**Sex**	F	16	15	1.00	9
	M	27	27		15
**AML onset**	De novo	38	34	0.38	17
	Secondary	5	8		7
**WBC (10** ^**9**^ **/L)**	(Min, Max), Median	(1.3, 263.0), 33.2	(1.4, 274.0), 19.1	0.62	(1.6, 120.2), 28.6
**Percent Health at 15 mins**	(Min, Max), Median	(26.0, 87.2), 52.2	(31.8, 86.3), 60.0	0.077	(29.8, 79.76), 50.4

a) CR = complete response; CRi = complete response with incomplete peripheral blood recovery; CCR1 = CR/CRi with duration > 1 yr; RD = resistant disease.

b) P-value for comparison of BM Training and BM Validation Analysis Sets.

c) Includes patients with known karyotype of indeterminate risk classification.

**Table 4:** Patient/Clinical Characteristics of the PB Training and Validation Analysis Sets.

Patient/Disease Characteristics	Subgroups	PB Training Analysis Set (n = 57)	PB Validation Analysis Set (n = 53)	P
**Response to induction therapy**	RD	14	10	0.87
	CR/CRi without CCR1	29	29	
	CCR1	14	14	
**Age (Years)**	(Min, Max), Median	(56.8, 83.9), 68.3	(59.0, 82.0), 66	0.17
**Cytogenetic risk group**	Better	7	6	0.99
	Intermediate	31	30	
	Poor	8	8	
	Missing	11	9	
**FLT3 ITD**	Mutant	16	17	0.68
	Wildtype	41	36	
**Sex**	F	33	20	0.038
	M	24	33	
**AML onset**	De novo	52	43	0.17
	Secondary	5	10	
**WBC (10** ^**9**^ **/L)**	(Min, Max), Median	(2.2, 298.0), 26.7	(4.7, 274), 21.9	0.28
**Percent Health at 15 minutes**	(Min, Max), Median	(27.9, 83.9), 60.6	(28.7, 83.1), 59.3	0.83

**Table 5:** Baseline Characteristics for SWOG Patients: SCNP-Evaluable vs. SCNP-Nonevaluable.

Patient/Disease Characteristics (SWOG)	Subgroups	SCNP-Evaluable (n = 213)	SCNP-Nonevaluable (n = 53)	P	Total (n = 266)
**Response to induction therapy**	RD	39	7	0.64	46
	CR/CRi without CCR1	67	18		85
	CCR1	39	13		52
	Fatal Induction Toxicity or Early Death	68	15		83
**Age (Years)**	(Min, Max), Median	(57, 88), 68	(56, 81), 70	0.71	(56, 88), 68
**Cytogenetic risk group**	Better	16	1	0.31	17
	Intermediate	121	30		151
	Poor	34	7		41
	Unknown	42	15		57
**Sex**	F	94	23	1.00	117
	M	119	30		149
**AML onset**	De Novo	162	45	0.20	207
	Secondary	51	8		59
	Unknown	0	0		0
**WBC (10** ^**9**^ **/L)**	(Min, Max), Median	(0.7, 298.0), 22.0	(0.8, 243.0), 41.1	0.09	(0.7, 298.0), 24.9
**Overall Survival (days)**	(Min, Max), Median	(1, 4866), 217	(1, 3799), 246	0.39	(1, 4866), 225

**Table 6:** Baseline Characteristics for ECOG Patients: SCNP-Evaluable vs. SCNP-Nonevaluable.

Patient/Disease Characteristics (ECOG)	Subgroups	SCNP-Evaluable (n = 24)	SCNP-Nonevaluable (n = 26)	P	Total (n = 50)
**Response to induction therapy**	CR/CRi	12	14	0.01	26
	Fatal Induction Toxicity or Early Death	0	6		6
	RD	12	6		18
**Age (Years)**	(Min, Max), Median	(57, 80), 69	(61, 76), 68	0.32	(57, 80), 68
**Cytogenetics Risk Group**	Intermediate	10	13	0.81	23
	Poor	5	4		9
	Unknown	9	9		18
**Sex**	F	9	15	0.17	24
	M	15	11		26
**AML Onset**	De Novo	17	18	1.00	35
	Secondary	7	7		14
	Unknown	0	1		1
**Pre-Induction WBC (10** ^**9**^ **/L)**	(Min, Max), Median	(1.6, 120.2), 28.6	(2.7, 107.0), 46.5	0.36	(1.6, 120.2), 33.9
**Overall Survival (days)**	(Min, Max), Median	(39, 2227), 231.5	(2, 1825), 223	0.52	(2, 2227), 227

### Model Building

For the 74 SWOG patients randomized to the Training Analysis Set, model building based on clinical prognostic factors universally known at the time of diagnosis (DX_CLINICAL 1_) was attempted but no significant predictive model could be built and therefore, no DX_CLINICAL1_ model was locked for validation. By contrast, using as inputs of both clinical prognostic factors (e.g. age, white blood cell count, blast percentage, etc.) and cytogenetic and molecular markers the predictive model DX_CLINICAL2_ achieved an out-of-bag estimated AUROC of 0.63 (see also S4 Methods).

For DX_SCNP_ predictive models, the functional measurement of induced apoptosis at 24 hours (Ara-C+Daunorubicin-induced c-PARP readout) was featured in all 5 models selected for verification based on performance indicating that the measurement of the change in the intra-cellular levels of c-PARP in the total blast population (after excluding Amina Aqua positive cells (i.e. necrotic cells) is a robust indicator of *in vivo* response to therapy (Table 1). The prediction accuracy, measured as AUROC, for these models (which were chosen based on modeling on PB training samples) when applied to the BM Training Analysis Set was similar to prediction accuracy for the classifier trained using BM training analysis set, justifying pursuing a single classifier for validation on both tissue types (Table 1). Thus, these 5 models were applied to the BM Verification Analysis Set and a final selected model was refined further using the BM and PB Training Analysis Set to create the final DX_SCNP_ classifier (Table 2) which was a logistic regression model with two nodes including Ara-C+Daunorubicin-induced c-PARP readout and CD34+ U_u._ The first node (Ara-C+Daunorubicin-induced c-PARP readout) is a measure of apoptosis induced by the drug treatment among blast cells that have not yet undergone necrosis. The second node, although not directly a measure of apoptosis, measures the remaining fraction of the CD34+ cell subset in the blast population after in vitro exposure to AraC+Daunorubicin (Table 2). The optimism-adjusted estimate of the AUROC for this predictor was 0.81 in the BM Training Analysis Set and 0.88 in the PB Training Analysis Set. This locked SCNP classifier, with all parameters fixed, was then applied to the BM Verification Analysis Set to estimate its true performance in an independent data set with resulting AUROC of 0.76, p = 0.01, 95% CI = (0.52, 0.91).

### Classifier Performance: BM Validation Analysis Set

**Fig 7:**
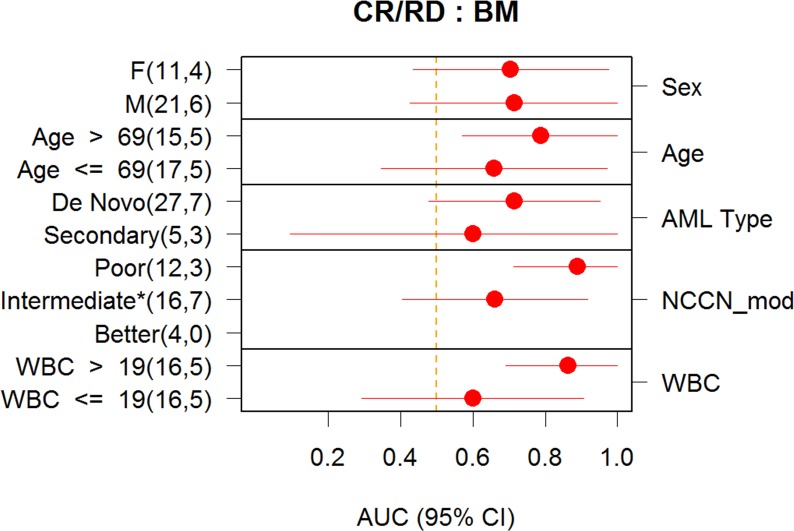
Performance of Classifier in Subgroups (BM). Prediction accuracy of DX_SCNP_ in various subgroups in the BM Validation Analysis Set. For age and WBC, the subgroups were defined by thresholding at the median value. For all samples cytogenetic risk was determined using NCCN 2013 guideline criteria. Similarly to what is done in clinical practice, patients with unknown cytogenetics were imputed as intermediate risk cytogenetics. The point estimate of accuracy measured by AUROC and confidence intervals (Delong method) are also shown.

The DX_SCNP_ classifier was validated as a predictor of CR/CRi in the BM Validation Analysis Set, with AUROC of 0.72, p = 0.02, 95% CI = (0.51, 0.87). In contrast, the DX_CLINICAL2_ classifier did not show a significant association with response to induction therapy in either the BM Verification Analysis Set (AUROC = 0.61, p = 0.18) or the BM Validation Analysis Set (AUROC = 0.53, p = 0.38). Furthermore, analysis was conducted to assess if DX_SCNP_ provided information for prediction of response that is independent of the DX_CLINICAL2_. The predictions from DX_CLINICAL2_ and DX_SCNP_ were both included (i.e., controlling for each other) in a combined logistic regression model for response. If predictions from DX_SCNP_ are redundant to those from DX_CLINICAL2_, a non-significant p-value is expected for the coefficient of DX_SCNP_ in the combined model. However, the SCNP classifier was still significant in predicting response in the BM Validation Analysis Set (p-value for DX_SCNP_ when controlling for DX_CLINICAL2_ = 0.03) from this analysis, showing that DX_SCNP_ may provide information that is independent from that provided by currently used prognostic markers. While the small sample sizes do not permit definitive comparisons of classifier accuracy between clinical subsets, the accuracy of predictions from DX_SCNP_ in BM sample subsets defined by several clinical characteristics are shown in Fig 7.

### Classifier Performance: PB Validation Analysis Set

**Fig 8:**
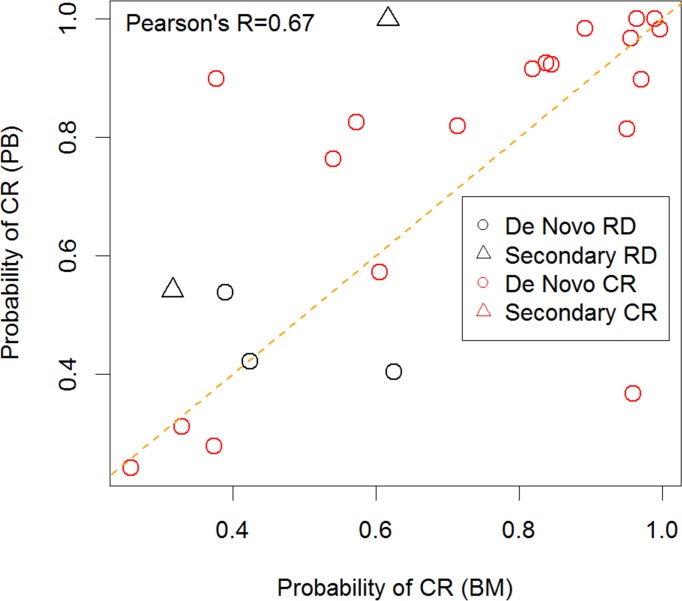
Comparison of predictions between paired PB and BM samples. Predicted probability of CR for BM and PB samples from donors with SCNP data with paired samples in the validation set. Denovo vs secondary AML subtypes are noted in the inset. A majority of the predictions were concordant between the tissues types of de novo. Of note, two RD donors that were discordant are secondary AML.

When the DX_SCNP_ classifier was applied to the PB Validation Analysis Set it did not accurately predict induction response (AUROC = 0.53, p = 0.39). A pre-specified subgroup analysis was performed for those with *de novo* AML vs. secondary AML at diagnosis since these subtypes have marked differences in clinical outcome [12], [13], [14], [15], [16] and data on a limited number of samples had previously shown that PB AML blasts in secondary AML have different signaling profiles than BM blasts [20]. Only three patients with secondary AML had paired PB and BM, precluding any useful analysis of concordance between the tissue types in this subgroup. However, in the *de novo* subgroup, DX_SCNP_ was a significant predictor of induction response in both PB and BM samples (Table 7). The correlation coefficient (Pearson’s R) was 0.67 when comparing predicted probability of CR for BM and PB samples among patient patients with both tissue types (Fig 8), with the predictions being concordant for a majority of the donors. However, the two donors with secondary AML that had paired samples were discordant. Further, among patients with *de novo* AML having both BM and PB samples, the values of DX_SCNP_ were correlated (Pearson’s R = 0.7) and had similar predictive value for the two sample types (AUROC = 0.71, p = 0.044, 95% CI = (0.50, 0.88) for the BM samples and AUROC = 0.79, p = 0.02, CI = (0.62–0.92) for PB samples). Given the small number of secondary AML donors that were RD in the training set, this difference in performance accuracy was not fully appreciated in the training phase.

**Table 7:** Prediction accuracy of DX_SCNP_ in BM and PB Validation Analysis subsets defined by AML Type (De Novo vs. Secondary) and availability of samples for both tissue types.

Sample Set	n (CR)	n (RD)	AUROC (95% CI)
BM	32	10	0.72 (0.53–0.91)
PB	43	10	0.53 (0.31–0.75)
De Novo BM	27	7	0.71 (0.48–0.95)
De Novo PB	38	5	0.79 (0.64–0.95)
Paired De Novo BM	19	3	0.74 (0.53–0.95)
Paired De Novo PB	19	3	0.79 (0.60–0.98)
Paired BM	19	5	0.76 (0.56–0.96)
Paired PB	19	5	0.65 (0.34–0.96)

### Response Prediction Using Amine Aqua

Measurement of the overall apoptotic capacity of single blasts was found to be a major component of all five candidate classifiers and was present in the final locked classifier. To determine whether a simple measure of cell viability using an exclusion dye such as amine aqua after incubation of the AML samples with Ara-C/daunorubicin for 24 hours could accurately predict response to induction therapy change in levels of in vitro cell death as measured by U_u_ metric for amina aqua was tested for association with clinical response. Results from this exercise showed that a simple measure of induced cell death at 24 hrs lacked the resolution to predict response to induction therapy (AUROC = 0.53, p = 0.37).

## Discussion

In the current study, quantitative measurement of intracellular signaling pathways in leukemic blasts was used to develop a predictor of response to induction therapy in elderly AML patients (defined in this study as >55 years old). In the patients studied, this predictor’s association with response was independent from that of currently used clinical and molecular variables. The process of classifier development was rigorous and followed the step-wise approach recommended by regulatory bodies consisting of a Training phase, followed by a Verification and a Validation phases in independent sample sets. This study confirms the accuracy and reproducibility of the assay platform and adds to the results of a previous study in BM samples from pediatric patients with AML [9], [30] in which a similarly developed classifier was shown to be an accurate predictor of induction response (AUROC = 0.70, p = 0.02), particularly in cytogenetically intermediate-risk pediatric patients (AUROC = 0.88, p = 0.002).

Traditionally, age, WBC count at diagnosis [31] and cytogenetics (the latter not always available at diagnosis, particularly at community and non-academic treatment settings [32], are the primary prognostic factors for induction treatment response in AML. Genetic factors such as the presence of *FLT3* ITD, CEBPα, and *NPM1* mutations, which have been incorporated into NCCN guidelines, provide additional prognostic information mostly useful for post-induction treatment planning (i.e. consolidation therapy). More recently, predictive models, such as a web-based application that uses standard clinical and laboratory values (e.g., body temperature, hemoglobin, age at diagnosis, platelets, *de novo* vs. secondary AML) and cytogenetic and molecular risk factors to generate an overall prognostic score, have been shown to have a significant association with induction response [5], [33].

In the current study the performance of an SCNP-based classifier was assessed in parallel against intra-study developed models which used only clinical (DX_CLINICAL1_) or both clinical and molecular parameters (DX_CLINICAL2_) as classifier inputs. The study design allowed for a descriptive comparison in the same patient population of the different classifiers’ performance. After controlling for clinical and genetic variables, the results supported the independence of the prognostic information provided by the SCNP-based classifier from that of traditional clinical and molecular markers [10]. Based on the independence of information provided by the SCNP and the Clinical classifiers, future efforts to train and validate models that include both kind of variables are certainly warranted.

Unfortunately, some of the inputs required to perform the risk score developed by Krug and colleagues (e.g. body temperature) were not available in our data set making it impossible to compare results from the web-based predictor of induction response [5] to the DX_SCNP_ classifier in our study.

Overall, our findings confirm the value of the SCNP-assay classifier, which can assess the functional effects of downstream multiple genetic and epigenetic molecular alterations.

Although the breadth of biology investigated in the training phase of the study included many signaling nodes in multiple pathways believed to be important in the leukemogenesis process and response to chemotherapy (e.g. cell survival, proliferation, DNA damage response, apoptosis pathways), the final locked and validated DX_SCNP_ classifier incorporated just two signaling nodes that assessed the functional capacity of the intracellular apoptosis pathway in the total blasts and the proportional reduction of CD34+ cells upon treatment in response to in vitro treatment with AraC and Daunorubicin). Of note, the cell-signaling based classifier developed in a pediatric AML population referenced above [9] included as input three signaling nodes measuring functional apoptosis, PI3 kinase, and proliferation pathways (i.e. etoposide induced c-PARP, FLT3L-induced p-S6, and Thapsigargin induced p-Erk). The presence of functional apoptosis in both the elderly and pediatric AML classifier is not an unexpected finding from a biologic point of view, considering that both classifiers were trained to predict remission induction, as defined by a reduction of BM AML blasts to less than 5%. However, it is important to note that a simple determination of cell death using amine aqua after incubation in vitro with chemotherapy agents did not correlate with response to induction chemotherapy (AUROC = 0.53, p = 0.37). The SCNP functional readout of apoptosis, in which dead cells are excluded by gating out cPARP positive (i.e., apoptotic) leukemic blast cells prior to analysis seems to better capture the ultimate results of positive and negative signals determining intrinsic leukemic cell survival capacity. In addition, the presence of PI3K and MAPK pathways read outs as input in the pediatric classifier only suggests that differential biology might be at the basis of AML primary chemotherapy resistance in the two age groups (thus needing different therapeutic approaches to overcome resistance).

Several methodological considerations and limitations need to be considered in interpreting these results. First, this study used cryopreserved samples (prospectively collected during the clinical trials) from biorepositories, rather than fresh samples. While this approach is efficient since it allows for batch analysis of large numbers of samples for which clinical annotations have already been collected, it raises concerns about the applicability of results to clinical settings (in which fresh samples will be used); and about the potential to introduce patient selection bias in the analysis, which could limit the generalizability of the classifier to different patient populations.

Previous studies have shown high correlation between SCNP readouts in paired fresh and cryopreserved aliquots of the same AML samples [34], suggesting it is likely that the SCNP-based classifier will have the same accuracy and reproducibility when applied to fresh samples [9], [34]. In addition stability data on PB and BM samples showed that the majority of fresh samples shipped at room temperature and received by the laboratory within 48 hours are suitable for reliable testing, i.e. the clinical assay will not suffer of the significant samples loss due to pre-analytic manipulations as experienced with the cryopreserved samples in this study. The predictive value of this specific classifier, when applied to fresh samples, remains to be confirmed in a prospective clinical trial.

Regarding potential selection bias, patients were selected on the basis of specimen availability and, as expected, evaluable patients had relatively higher WBC counts and blast percentages when compared to the non-evaluable patients (S2 Table). Accuracy of predictions from DX_SCNP_ in BM sample subsets defined by clinical characteristics is shown in Fig 7. While the small sample sizes do not permit definitive comparisons of classifier accuracy between subsets, it is notable that the AUROC for DX_SCNP_ is somewhat higher for patients with WBC count greater than the median of 19x10^9^/L: 0.86 vs 0.60. The difference in prediction accuracy between subgroups defined by Sex and Age are even less significant. Prospective studies will be required to evaluate the role of SCNP-based predictors in fresh cells from a more representative population of newly diagnosed patients.

The purpose of our study was to identify and validate a predictive classifier for response to standard induction therapy using as inputs intracellular functional pathway readouts. Despite the heterogeneity of the patient population studied (i.e. samples obtained from patients enrolled on different studies that were conducted over greater than a 10 year span) the SCNP classifier that was verified and validated (verification AUROC of 0.76, p = 0.01 and validation AUROC = 0.72, p = 0.02) was quite robust. These data underscore that the biology identified and measured using SCNP is crucial to AML blast in vivo chemosensitivity and are in alignment with data by Vo and colleagues that demonstrate the relative priming of AML blasts toward apoptosis is associated with clinical outcomes [35]. Of note, in those studies, the cryopreservation and thaw process, similar to our experience with SCNP, did not change the priming of the leukemic cells. The predictive ability and clinical utility of BH3 profiling, and how it compares to SCNP is currently unknown.

Compared with the BM Verification and Validation Analysis Sets, the BM Training Analysis Set had a lower percentage of patients with secondary AML, which was not a stratification factor during randomization (12% in Training vs. 29% in Verification and 19% in Validation). Although these differences were not statistically significant, likely due to the small sample size, the differences in biology of *de novo* and secondary AML could have affected model performance characteristics during the Verification and Validation phases, particularly in the PB Validation Analysis Set (PB prediction of response: AUROC 0.53, p = 0.39). When paired (from the same patient) BM and PB samples were grouped by AML onset (*de novo* vs. secondary), the SCNP classifier scores were concordant between BM and PB in the *de novo* subset (Pearson R = 0.7). Furthermore, DX_SCNP_ was a reliable (Table 7) predictor of response in the *de novo* Validation subgroup, (AUROC 0.71, p = 0.044, and AUROC 0.79, p = 0.02, for the BM and PB Validation Analysis Subsets, respectively). For the 10 patients with secondary AML in the PB Validation Analysis Subset, 5 had outcome of RD and all 5 were predicted incorrectly by DX_SCNP_ as compared to 2 RD donors in the PB training set. These findings are consistent with prior data, which indicated that the underlying biology of secondary AML is different from that of *de novo* AML [7] and that leukemic cell populations present in BM may have different characteristics from those found in PB.

In sum, the results of this study confirm the ability of quantitative SCNP testing using functional flow cytometry to predict induction response in elderly AML patients. The assay provides accurate, independent data on disease biology and has the potential to inform treatment choices by allowing patients to avoid harmful treatment when it is likely futile, while offering the opportunity for those patients to consider enrollment in clinical trials evaluating new targeted and less intensive regimen as first line treatment. Prospective studies in adults and children are planned that move the Dx_SCNP_ classifier prospectively into the clinical setting to confirm its feasibility and value as a provider of independent and actionable information for consideration prior to initiating therapy. Such validation may allow the development of a taxonomy of response to induction therapy and a more comprehensive understanding of the variegated blast-cell signaling patterns seen in de novo vs. secondary AML. A parallel SCNP study, initially retrospective and latter prospective, is planned to develop classifiers for prediction of disease relapse in patients who achieve CR after induction therapy to guide individualized consolidation treatments.

## Supporting Information

S1 Fig SCNP Node Metrics Diagram. (DOCX)

S1 Inputs Node Assay Panel. (DOCX)

S1 Methods Randomization. (DOCX)

S2 Methods Variable Selection. (DOCX)

S3 Methods Imputation of clinical data for the development of DX_CLINICAL1_ and DX_CLINICAL2._
(DOCX)

S4 Methods Clinical predictor DX_CLINICAL2._
(DOCX)

S5 Methods Validation Methods. (DOCX)

S1 MiFlowCyt Report (DOCX)

S1 Table SWOG and ECOG Treatment Study Details. (DOCX)

S2 Table Baseline characteristics of patients on SWOG trials. (DOCX)

S3 Table SCNP node-metrics. (DOCX)
