# Supplementary figures and images for: Cell Signaling-Based Classifier Predicts Response to Induction Therapy in Elderly Patients with Acute Myeloid Leukemia
Source: PLoS One. 2015 Apr 17;10(4):e0118485. doi: 10.1371/journal.pone.0118485 (PMC4401549; doi:10.1371/journal.pone.0118485)

**S1 Figure: SCNP Node Metrics Diagram**
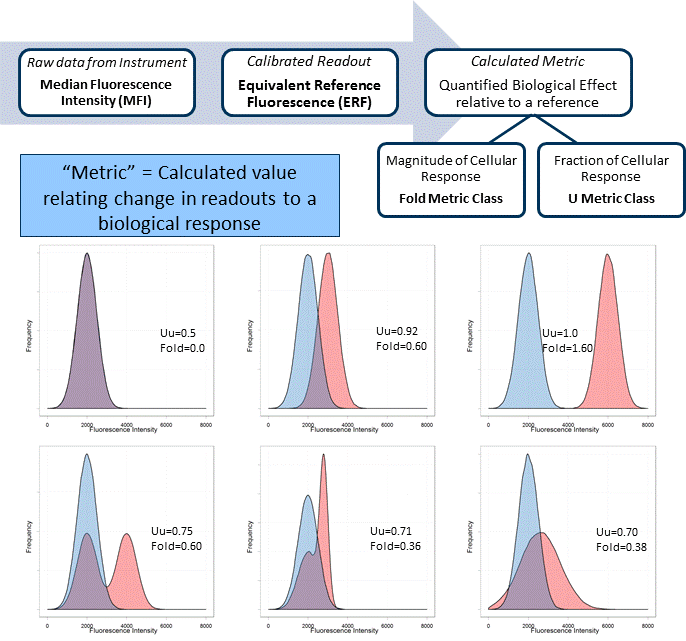

Supplement: S1 Fig — (DOCX) [file pone.0118485.s001.docx]
