# Supplementary material for: Cell Signaling-Based Classifier Predicts Response to Induction Therapy in Elderly Patients with Acute Myeloid Leukemia
Source: PLoS One. 2015 Apr 17;10(4):e0118485. doi: 10.1371/journal.pone.0118485 (PMC4401549; doi:10.1371/journal.pone.0118485)
Supplement: S1 Inputs — (DOCX) [file pone.0118485.s002.docx]

## S1 Inputs: Inputs for the SCNP-Based Classifier: Node Assay Panel

The full list of signaling nodes in the assay panel for this study (n=53) is shown in Table A. A signaling node is defined as a combination of a modulator with an intracellular read out. Approximately 2x10^6^ cells were required to run the full planned panel of signaling nodes (53 nodes). However for some patients, due to lower total number of viable cells in the sample post thaw and ficoll, SCNP data was collected for only a subset of the planned nodes. In order to avoid data imputation, SCNP data from the 35 highest priority nodes (see Supplemental Table S3) was used to develop the SCNP-based classifier.

After completion of the SCNP assay, the following pre-specified criteria for determining evaluable BMMC and PBMC samples were applied: 1) a minimum of 25% healthy cells measured as the percentage of cPARP negative cells in the viable leukemic blast population, 2) a minimum of 500 viable healthy cells per well in the leukemic cell gate, 3) SCNP data available for the 35 highest priority nodes and 4) the absence of any technical assay deviation. SCNP readout data were stored on a secure restricted-access server at Nodality.

**Table A: Node Assay Panel^1^**

|  | **Modulator*** | **Duration of Modulator treatment** | **Lineage & gating markers** | **Intracellular Readout** |
| --- | --- | --- | --- | --- |
| 1 | Phenotyping | N/A | CD38, CD135, CD15, CD34, CD11b-, CD117, CD45 | (None) |
| 2 | AF | 15 min | AA, CD45, CD34 | (None - AF background) |
| 3 | UM^1^ | 15 min | AA, CD45, CD34, cPARP | (p-Chk2,P21,cPARP) |
| 4 | UM | 240 min | AA, CD45, CD34, cPARP | (p-Chk2,P21,cPARP) |
| 5 | UM | 1440 min | AA, CD45, CD34, cPARP | (p-Chk2,P21,cPARP) |
| 6 | Ara-C+DNR | 1440 min | AA, CD45, CD34, cPARP | (p-Chk2,P21,cPARP) |
| 7 | Ara-C+DNR+CSA | 1440 min | AA, CD45, CD34, cPARP | (p-Chk2,P21,cPARP) |
| 8 | UM | 15 min | AA, CD45, CD34, cPARP | (p-CREB, p-Erk, p-S6) |
| 9 | PMA | 15 min | AA, CD45, CD34, cPARP | (p-CREB, p-Erk, p-S6) |
| 10 | UM | 15 min | AA, CD45, CD34, cPARP | (p-Akt, p-Erk, p-S6) |
| 11 | FLT3L | 15 min | AA, CD45, CD34, cPARP | (p-Akt, p-Erk, p-S6) |
| 12 | SCF | 15 min | AA, CD45, CD34, cPARP | (p-Akt, p-Erk, p-S6) |
| 13 | UM | 15 min | AA, CD45, CD34, cPARP | (p-Stat1, p-Stat3, p-Stat5 |
| 14 | IL-27 | 15 min | AA, CD45, CD34, cPARP | (p-Stat1, p-Stat3, p-Stat5) |
| 15 | G-CSF | 15 min | AA, CD45, CD34, cPARP | (p-Stat1, p-Stat3, p-Stat5) |
| 16 | AF | 1440 min | CD45, CD34 | (None- AF background) |
| 17 | Etoposide | 1440 min | AA, CD45, CD34, cPARP | (p-Chk2,P21,cPARP) |
| 18 | Thapsigargin | 15 min | AA, CD45, CD34, cPARP | (p-CREB, p-Erk, p-S6) |

^1^UM=unmodulated
