# Supplementary material for: Cell Signaling-Based Classifier Predicts Response to Induction Therapy in Elderly Patients with Acute Myeloid Leukemia
Source: PLoS One. 2015 Apr 17;10(4):e0118485. doi: 10.1371/journal.pone.0118485 (PMC4401549; doi:10.1371/journal.pone.0118485)
Supplement: S1 Methods — (DOCX) [file pone.0118485.s003.docx]

## S1 Methods: Randomization

After receiving the list of evaluable samples, the SWOG Statistical Center identified the evaluable SWOG patients (i.e. patients having an evaluable sample: BM, PB or both), who were then randomized approximately 1:1 between the Training and Validation Sets. The Pocock-Simon method was used to ensure near balance for each of the following variables :

1. Induction response: CR/CRi duration < 1 year vs. CR/CRi duration > 1 year vs. resistant disease (RD) vs. fatal induction toxicity (FIT) or early death (ED) in the absence of FIT
2. Pre-treatment specimen availability: BM only vs. PB only vs. both
3. Cytogenetic risk group: core binding factor vs. cytogenetically normal vs. poor risk (defined by total/partial deletion of 5q and/or 7q) vs. unknown vs. all others
4. Parent trial treatment arm: SWOG-9031 arm 1 vs. SWOG-9031 arm 2 vs. S9333 arm 1 vs. S0112 vs. S0301 (see Supplemental Table S2 for treatment regimens)
5. FLT3-ITD mutational status (BM): Yes vs. No vs. unknown
6. FLT3-ITD mutational status (PB): Yes vs. No vs. unknown
7. BM specimen evaluability*: Data available for all proteomic readouts vs. data available for minimum required proteomic readouts vs. not evaluable
8. PB specimen evaluability*: Data available for all proteomic readouts vs. data available for minimum required proteomic readouts vs. not evaluable

*Randomization was applied to patients, not specimens; therefore, each patient was stratified on the basis of pretreatment BM specimen evaluabilty, and on the basis of pretreatment PB specimen evaluability.

After SWOG uploaded the patient randomization list to the secure website, Nodality isolated raw data (i.e., FCS files/gating files) for the validation set on a restricted-access server location. Nodality staff then calculated node-metrics for the Training Set only.
