# Supplementary material for: Cell Signaling-Based Classifier Predicts Response to Induction Therapy in Elderly Patients with Acute Myeloid Leukemia
Source: PLoS One. 2015 Apr 17;10(4):e0118485. doi: 10.1371/journal.pone.0118485 (PMC4401549; doi:10.1371/journal.pone.0118485)
Supplement: S2 Methods — (DOCX) [file pone.0118485.s004.docx]

## S2 Methods: Variable Selection

The form of the relationship between node-metrics and binary outcomes (response to induction therapy) were investigated using random forest (Breiman, 2003 and Liaw and Wiener, 2002), penalized logistic regression (Goemann, 2010), traditional logistic regression with natural spline functions (Harrell, 2001), and loess regression (Harrell, 2001). Logistic regression, using natural spline transformations, and loess regression, when applied, helps identify non-linear functional forms and suggest transformations that strengthen the association with the outcome. Functional forms were also investigated by examining partial dependence plots (random forest) and model residuals (logistic and proportional hazards regression).

The Random Forest method was used for identifying a subset of variables whose relationship with the outcome can be represented as a step function (monotonic or non-monotonic) or involves an interaction with other variables. Penalized logistic regression was used for identifying subsets of variables that have strong linear relationships with the outcome.

Node-metrics that were ranked low (i.e., weak association with the outcome of interest) by both random forest and penalized logistic regression methods, had a low rank-order correlation (e.g. Spearman correlation coefficient or Somers’ D) with the outcome, and/or which did not exhibit a functional form that could be modeled with a simple transformation (i.e. using few degrees of freedom), were excluded from further consideration as predictors of that outcome.

In an effort to further reduce the dimensionality of the modeling effort (i.e. the number of node-metric candidates under consideration) for a given outcome, measures of association were contrasted for modulator-antibody combinations (nodes) measured by alternative metrics, after appropriate transformations were applied. If one metric yielded consistently stronger relationships with the outcome compared to an alternative, the alternative metric was excluded from further consideration.

The impact of the following factors on the strength and form of the relationship between each node-metric and the outcome was investigated: percent health, cell maturity, cytogenetics, FLT3R ITD mutational status (binary and continuous), and completion of induction therapy. The strength and form of the relationship between each node-metric and the induction response outcome were investigated separately in the set of all evaluable patients and in the subset excluding induction deaths. If relationships between node-metrics and outcomes were sufficiently different, as a function of any such factors, those factors were accounted for through adjustment of node-metric signals, incorporation of those factors into the modeling process, or development of separate models**.** After appropriate transformations were identified and apparently poor predictors and/or inferior metrics were excluded from further consideration, the remaining candidates were evaluated as predictors of the outcome in different multivariate models.
