# Supplementary material for: Cell Signaling-Based Classifier Predicts Response to Induction Therapy in Elderly Patients with Acute Myeloid Leukemia
Source: PLoS One. 2015 Apr 17;10(4):e0118485. doi: 10.1371/journal.pone.0118485 (PMC4401549; doi:10.1371/journal.pone.0118485)
Supplement: S3 Methods — (DOCX) [file pone.0118485.s005.docx]

## S3 Methods: Imputation of clinical data for the development of DX_CLINICAL1_ and DX_CLINICAL2_

Among the variables included in the development of DX_CLINICAL1_ and DX_CLINICAL2_, data were not available for 1-6% of the patients for following variables: absolute blast count, percentage of blasts, monocytes, neutrophils, FLT3 ITD status, NPM1 mutational status, race, hemoglobin, and/or platelet count data. The following process was followed to impute the missing data:

1. Missing absolute blast count was estimated from WBC values using a linear function. The linear function was obtained by regressing absolute blast count against WBC for those donors for whom both values were available.
2. Missing percentage blasts value were then computed as

$$\text{\% blast = }\frac{\text{100*(absolute blast count)}}{\text{WBC}}$$

1. Percentage monocytes (where possible) were computed as

1. Absolute monocyte count was then computed as

$$\text{abs.monocytes = }\frac{\text{(\%monocytes)*(WBC)}}{100}$$

1. Where possible, similar strategy shown in items c and d was employed to impute missing data for percentage of neutrophils and absolute neutrophil counts

$$\text{abs.neutrophils = }\frac{\text{(\%neutrophils)*(WBC)}}{100}$$

1. FLT3 ITD mutation status for all donors with missing data was set to wild type (WT)
2. Similarly NPM1 mutation status for all donors with missing data was set to WT
3. The remaining missing data (a maximum 3% for any of the variable) was imputed using k-Nearest Neighbhor (KNN) method implemented in *imputation* library in R software. This method was applied to the data after the variables WBC, neutrophil, blast, monocyte, absolute neutrophil count, and platelet counts have been transformed to a log scale, followed by scaling of the data to zero mean and unit variance (z-transform). The variables included in the k-nearest neighbor computation are: age, percentage of blast, monocytes, neutrophils, absolute counts of neutrophils, blast, monocytes, platelet count, hemoglobin and FAB (categorized as 0 or 1 for mature and immature).
