# Supplementary material for: Cell Signaling-Based Classifier Predicts Response to Induction Therapy in Elderly Patients with Acute Myeloid Leukemia
Source: PLoS One. 2015 Apr 17;10(4):e0118485. doi: 10.1371/journal.pone.0118485 (PMC4401549; doi:10.1371/journal.pone.0118485)
Supplement: S4 Methods — (DOCX) [file pone.0118485.s006.docx]

## S4 Methods: Clinical predictor DX_CLINICAL2_

## The clinical predictor DX_CLINICAL2_ was a logistic function of the form:

$$\text{DX}_{\text{CLINICAL2}}=\frac{e^{\chi^{'}\widehat{\beta}}}{\text{1+}e^{\chi^{'}\widehat{\beta}}}\text{ where }\chi^{'}\text{is the vector of input parameters and }\widehat{\beta}\text{ is the vector of estmated regression coefficients}$$

## Based on the N=74 patients in the Training Set, the predictor was defined as follows:

| **Input parameter** | **Estimated regression coefficient** |
| --- | --- |
| *χ*^0^: Intercept = 1 for all patients | 1.1165444 |
| *χ*^1^: Cytogenetic risk group = 1 for Poor Risk, 0 for all others | −0.9532412 |
| *χ*^2^: NPM1 mutation status = 1 for mutant, 0 for wildtype | 0.3518013 |
| *Χ*^3^: FLT3/NPM1 status = 1 for ITD/mutant, 0 for all others | 0.4051878 |

Note that the three included parameters are all dichotomous and define 6 possible values of DX_CLINICAL2_. The following table summarizes DX_CLINICAL2_ and the corresponding response rates in the Training and Validation Sets:

| **Cytogenetic**  **risk group** | **NPM1**  **status** | **FLT3-ITD** | **DX_CLINICAL2_** | **Training Set CR/N (%)** | **Validation Set CR/N (%)** |
| --- | --- | --- | --- | --- | --- |
| Poor risk | Mutant | ITD+ | 72% | 0/0 (---) | 2/2 (100%) |
|  | Mutant | ITD− | 63% | 0/0 (---) | 1/1 (100%) |
|  | Wildtype | Either | 54% | 4/10 (40%) | 6/9 (67%) |
| Any other | Mutant | ITD+ | 87% | 11/11 (100%) | 9/9 (100%) |
|  | Mutant | ITD− | 81% | 16/19 (84%) | 7/8 (88%) |
|  | Wildtype | Either | 75% | 25/34 (74%) | 31/43 (72%) |
