# Supplementary material for: Cell Signaling-Based Classifier Predicts Response to Induction Therapy in Elderly Patients with Acute Myeloid Leukemia
Source: PLoS One. 2015 Apr 17;10(4):e0118485. doi: 10.1371/journal.pone.0118485 (PMC4401549; doi:10.1371/journal.pone.0118485)
Supplement: S5 Methods — (DOCX) [file pone.0118485.s007.docx]

## S5 Methods: Validation Methods

Upon SWOG’s confirmation of receipt of the final, locked classifier, Nodality calculated the SCNP node-metrics for the SWOG Validation Analysis Set and the final predictions using Nodality-developed software (validated for this intended use). The final predictions were transferred to SWOG, who then provided the final clinical outcomes. The performance of the classifiers was evaluated independently at Nodality and at either SWOG or ECOG (for each group’s own respective datasets).
