# Supplementary material for: Cell Signaling-Based Classifier Predicts Response to Induction Therapy in Elderly Patients with Acute Myeloid Leukemia
Source: PLoS One. 2015 Apr 17;10(4):e0118485. doi: 10.1371/journal.pone.0118485 (PMC4401549; doi:10.1371/journal.pone.0118485)
Supplement: S1 MiFlowCyt Report — (DOCX) [file pone.0118485.s008.docx]

MIFLOWCYT Summary for

Cesano, A., et al., PLoS One, XXX.

MiFlowCyt Report - Cell Signaling-based Classifier Predicts Response to Induction Therapy in Elderly Patients with Acute Myeloid Leukemia

1. Table of Contents

[1. Table of Contents 2](#_Toc396988979)

[2. List of Abbreviations and Definitions of Terms 4](#_Toc396988980)

[3. Experiment Overview 4](#_Toc396988981)

[3.1. Purpose 4](#_Toc396988982)

[3.2. Keywords 5](#_Toc396988983)

[3.3. Experiment Variables 5](#_Toc396988984)

[3.4. Organization 5](#_Toc396988985)

[3.5. Primary Contact 5](#_Toc396988986)

[3.6. Dates During Which Study Was Conducted 5](#_Toc396988987)

[3.7. Conclusions 5](#_Toc396988988)

[4. Instrument Details 6](#_Toc396988989)

[4.1. Instrument Manufacturer 6](#_Toc396988990)

[4.2. Instrument Configuration and Settings 6](#_Toc396988991)

[5. Reagents Used in The Experiment 6](#_Toc396988992)

[5.1. Experimental Control Reagents 6](#_Toc396988993)

[5.2. Modulators 7](#_Toc396988994)

[5.3. Antibodies 7](#_Toc396988995)

[5.4. Summary of Modulators, Timing, and Cocktail Combinations 8](#_Toc396988996)

[5.5. Plate Layouts 9](#_Toc396988997)

[6. Quality Control Measures 11](#_Toc396988998)

[6.1.1. Rainbow Control Particles (RCP) 11](#_Toc396988999)

[6.1.2. Cell Lines 13](#_Toc396989000)

[7. Flow Sample/Specimen Details 14](#_Toc396989001)

[7.1. Sample/Specimen Material Description 14](#_Toc396989002)

[7.2. Sample Treatment(s) Description 14](#_Toc396989003)

[8. Data Analysis Details 14](#_Toc396989004)

[8.1. List-mode Data File 14](#_Toc396989005)

[8.2. Compensation Details 14](#_Toc396989006)

[8.3. Gating (Data Filtering) Details 15](#_Toc396989007)

[8.3.1. Gate Description 15](#_Toc396989008)

[8.3.1.1. P1 gate 16](#_Toc396989009)

[8.3.1.2. Healthy P1 gate 16](#_Toc396989010)

[8.4. Data Transformation Details and SCNP Metrics 16](#_Toc396989011)

[8.4.1. Node-Metrics Used in the Training and Validation Studies 18](#_Toc396989012)

[8.4.2. Gate Boundaries 18](#_Toc396989013)

[9. Final sample disposition table 19](#_Toc396989014)

[10. Relevant Experiment Information In Supplemental Materials 19](#_Toc396989015)

1. List of Abbreviations and Definitions of Terms

| Term | Definition/Explanation |
| --- | --- |
| DMSO | Dimethyl sulfoxide |
| ERF | Equivalent Number of Reference Fluorophores |
| FACS buffer | 1X PBS + 0.5% BSA with 0.05% NaN3 |
| FBS | Fetal Bovine Serum |
| FCS | Flow cytometry standard file |
| FSC | Forward scatter |
| GDM-1 | AML cell line |
| MFI | Mean fluorescence intensity |
| PBS | Phosphate buffered saline |
| PBS + 0.1% NaN3 | High-Purity (filtered) Phosphate buffered saline + Sodium Azide |
| PFA | Paraformaldehyde |
| RCP | Rainbow calibration particles |
| RPMI | RPMI 1640 – tissue culture medium |
| RS4;11 | ALL cell line. FLT3L responsive. |
| SCNP | Single cell Network Profiling |
| SSC | Side scatter |
| Thaw buffer | RPMI media + 60% FBS |
| Wash buffer | 1X PBS + 0.5% BSA without 0.05% NaN3 |
| WBC | White blood cell count derived from the AcT10 hematology instrument |
| WinList | Listmode analysis software used by Nodality (Verity Software House) |

1. Experiment Overview
   1. Purpose

To develop and validate a SCNP classifier (DXSCNP) for the prediction of response to Ara-C-based induction chemotherapy using bone marrow (BM) and peripheral blood (PB) samples from elderly patients with newly diagnosed AML.

- 1. Keywords

SCNP, Single Cell Network Profiling, AML, Acute Myeloid Leukemia, multiparametric flow cytometry

- 1. Experiment Variables

Experiments were performed on cryopreserved peripheral blood (PB) and bone marrow (BM) AML samples collected as part of SWOG Studies SWOG-9031, SWOG-9333, S0112 or S0301 and ECOG Studies E3993 and E3999). The GDM1 and RS4;11 cell lines served as positive controls for all assays performed.

Stained cells were acquired on standardized Becton Dickinson FACS Canto II flow cytometers. All reagents are specified below.

Refer to Experimental Details in Cesano, et al, for more details on experimental variables.

- 1. Organization

Nodality, Incorporated

170 Harbor Way, Suite 200, South San Francisco, CA 94080

- 1. Primary Contact

Santosh Putta, Vice President, Computational Sciences

santosh.putta@nodality.com

Data requests can be made by sending an email to data.request@nodality.com.

- 1. Dates During Which Study Was Conducted

The assay was conducted over a 9 week period with 2 batches per week and 28 samples per batch. A total of 435 samples (from 266 patients) were eligible for the study and were thawed, treated with modulators, stained, and analyzed using flow cytometry between September 19, 2011 and November 18, 2011. All gating was performed manually using the WinList software package (Verity Software House, Topsham, ME). Gating was completed December 14, 2011. Determination of evaluability was completed by December 14, 2011.

- 1. Conclusions

This study describes the training and validation of a classifier which uses inputs from multi-parametric analysis of intracellular signaling pathways to predict response to therapy in elderly AML patients. The results of this study confirm the ability of quantitative SCNP testing using functional flow cytometry to predict a clinical outcome such as induction response in elderly AML patients.

1. Instrument Details
   1. Instrument Manufacturer

All flow cytometry data were collected on three Becton Dickinson FACS CANTO II cytometers.

| Nodality Asset Tag | Manufacturer Serial Number | Installation Date (including filter sets) |
| --- | --- | --- |
| 00731 | V96300490 | 2 Oct 2009 |
| 00419 | V96300493 | 9 July 2008 |
| 00926 | V96300766 | 2 Feb 2010 |

- 1. Instrument Configuration and Settings

No alterations have been made to the flow cytometers with the exception of the following listed dichroic mirror/filter combinations. The optical paths are as shown below.

| 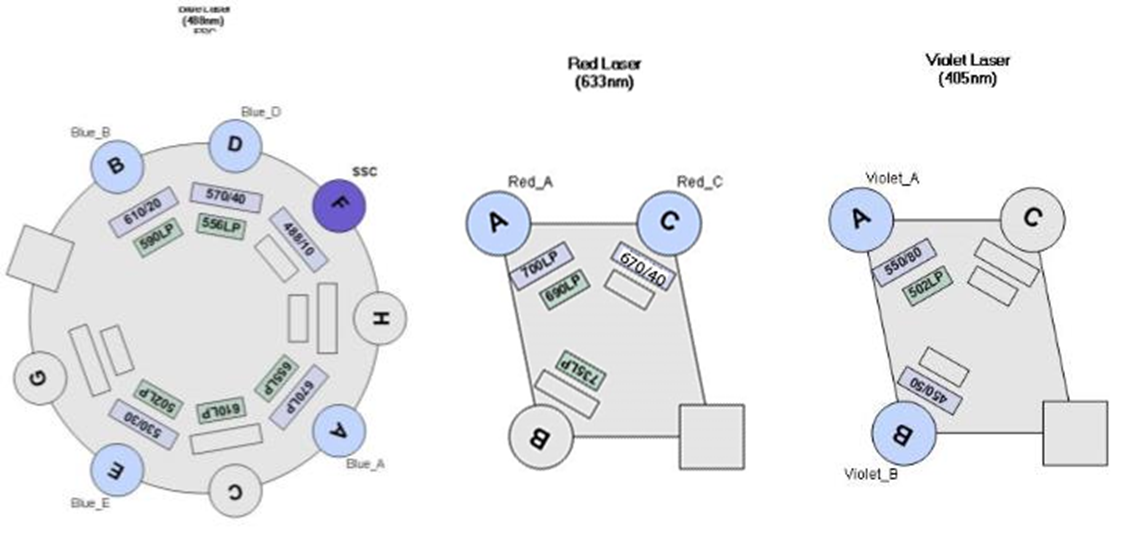 |
| --- |
| Optical path configuration of BD FACS Canto II cytometers used for this study, including filter sets. |

1. Reagents Used in The Experiment
   1. Experimental Control Reagents

The following control cell lines and RCP were used in this study

| **Name** | **Supplier** | **Catalog No.** |
| --- | --- | --- |
| 8 peak rainbow beads | Spherotech Inc. | RPC-30-5A |
| GDM1 cell line | ATCC | TIB-2627 |
| RS4;11 cell line | ATCC | TIB-1873 |

- 1. Modulators

The table below provides a listing of vendor and catalog information for the modulators used in in the study. Each modulator was qualified by identifying an intra-cellular readout and control sample/cell line that is expected to display induced signaling. The modulator was then titrated to identify optimal saturating concentration at which no further increasing in modulated signaling is observed.

| **Name** | **Supplier** | **Catalog Number** |
| --- | --- | --- |
| Ara C | Sigma | C1768 |
| Cyclosporin A | Calbiochem | 239835 |
| Daunorubicin | Sigma | D8809 |
| Etoposide | Sigma | E1383 |
| FLT3L | eBio | 14-8358-80 |
| G-CSF | R&D Systems | 214-CS |
| IL-27 | R&D Systems | 2526-IL |
| PMA | Sigma | P8139 |
| SCF | R&D Systems | 255-SC |
| Thapsigargin | Calbiochem | 586005 |

- 1. Antibodies

The following table provides vendor and catalog number information for each of the antibodies used in this study. Each of the lineage/gating marker antibodies was qualified by performing a serial titration of antibody concentrations using samples known to express cell subsets with positive and negative expression of the antibody. Similarly, each of the intra-cellular signaling antibodies was qualified by performing a titration using appropriate modulated and unmodulated control samples/cell lines (e.g. the p-S6 antibody was titrated against unmodulated as well as PMA modulated GDM-1 cells). The optimal antibody concentration was identified to maintain saturation and yield the optimal signal to noise ratio for gating antibodies or optimal evoked Log2Fold response for signaling antibodies.

| **Antibody/Conjugate Supplier, Manufacturing, Testing and Specification Documents** | | |
| --- | --- | --- |
| **Name** | **Supplier** | **Catalog No.** |
| CD117-APC | DAKO | C7244 |
| CD11b-PacBlue | Nodality | Original Supplier Catalog number;  conjugated at Nodality |
| CD135-PE | BD Biosciences | 558996 |
| CD15-Biotin* | BioLegend | 323016 |
| CD34-PE | BD Biosciences | 348057 |
| CD34-PerCP | BD Biosciences | 340666 |
| CD45-AF700 | Nodality | Original Supplier Catalog number;  conjugated at Nodality |
| cPARP-FITC | BD Biosciences | 558576 |
| cPARP-PacBlue | Nodality | Original Supplier Catalog number;  conjugated at Nodality |
| p-AKT-AF647 | CST | 2337 |
| p-CHK2-AF647 | CST | 2197 |
| p-CREB-PE | BD Biosciences | 558436 |
| p-ERK1/2-AF647 | BD Biosciences | 612593 |
| p-ERK1/2-PE | BD Biosciences | 612566 |
| p-S6-AF488 | BD Biosciences | 558438 |
| p-STAT1-AF488 | BD Biosciences | 612596 |
| p-STAT3-PE | BD Biosciences | 612569 |
| p-STAT5-AF647 | BD Bioscience | 612599 |

- 1. Summary of Modulators, Timing, and Cocktail Combinations

The table below shows the condition (combinations of modulator/inhibitor, modulation time, and the antibodies) in each well in which AML sample was plates. Following established SOPs at Nodality, the antibodies were combined into cocktails prior to starting of the experimental phase. Each cocktail, consisted of lineage or gating markers, common across multiple cocktails, as wells as intra-cellular signaling markers.

| **Modulator*** | **Modulator Concentration** | **Duration of Modulator treatment** | **Antibody Cocktail** | **Lineage & gating markers** | **Intracellular Readout** |
| --- | --- | --- | --- | --- | --- |
| Pheno |  | N/A | AML-15 | CD38, CD135, CD15, CD34, CD11b-, CD117, CD45 | None |
| AF |  | 15 min | AML-08 | AA, CD45, CD34 | None - AF background |
| UM |  | 15 min | AML-14 | AA, CD45, CD34, cPARP | p-Chk2, p21 |
| UM |  | 240 min | AML-14 | AA, CD45, CD34, cPARP | p-Chk2, p21 |
| UM |  | 1440 min | AML-14 | AA, CD45, CD34, cPARP | p-Chk2, p21 |
| Ara-C+  Daunorubicin | 500 ng/mL  100 ng/mL | 1440 min | AML-14 | AA, CD45, CD34, cPARP | p-Chk2, p21 |
| Ara-C+  Daunorubicin + Cyclosporin A | 500 ng/mL  100 ng/mL  2.5 µg/mL | 1440 min | AML-14 | AA, CD45, CD34, cPARP | p-Chk2, p21 |
| UM |  | 15 min | AML-03 | AA, CD45, CD34, cPARP | p-CREB, p-ERK, p-S6 |
| PMA | 400 nM | 15 min | AML-03 | AA, CD45, CD34, cPARP | p-CREB, p-ERK, p-S6 |
| UM |  | 15 min | AML-02 | AA, CD45, CD34, cPARP | p-Akt, p-ERK, p-S6 |
| FLT3L | 50 ng/mL | 15 min | AML-02 | AA, CD45, CD34, cPARP | p-Akt, p-ERK, p-S6 |
| SCF | 20 ng/mL | 15 min | AML-02 | AA, CD45, CD34, cPARP | p-Akt, p-ERK, p-S6 |
| UM |  | 15 min | AML-01 | AA, CD45, CD34, cPARP | p-Stat1, p-Stat3, p-Stat5 |
| IL-27 | 50 ng/mL | 15 min | AML-01 | AA, CD45, CD34, cPARP | p-Stat1, p-Stat3, p-Stat5 |
| G-CSF | 50 ng/mL | 15 min | AML-01 | AA, CD45, CD34, cPARP | p-Stat1, p-Stat3, p-Stat5 |
| AF |  | 1440 min | AML-11 | CD45, CD34 | None- AF background |
| Etoposide | 30 µg/mL | 1440 min | AML-14 | AA, CD45, CD34, cPARP | p-Chk2,P21,cPARP |
| Thapsigargin | 1 µM | 15 min | AML-03 | AA, CD45, CD34, cPARP | p-CREB, p-ERK, p-S6 |

*AF-autofluorescence; Pheno – phenotypic characterization cocktail; UM- unmodulated;

- 1. Plate Layouts

Samples were run in batches using 96-well plates. A total of 14 samples were processed per batch and two batches were performed on each experimental day. The plates corresponding to the functional readouts in signaling pathways included one row of cell line controls and 7 samples per plate as shown below, requiring two plates per batch. The apoptosis plates (4-hour and 24-hour) included one row of cell line controls and 14 donors per plate.

| Signaling Plate 1 | 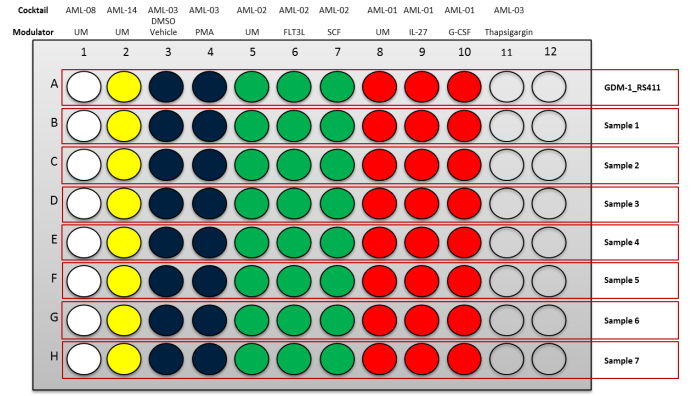 |
| --- | --- |
| Signaling Plate 2 | 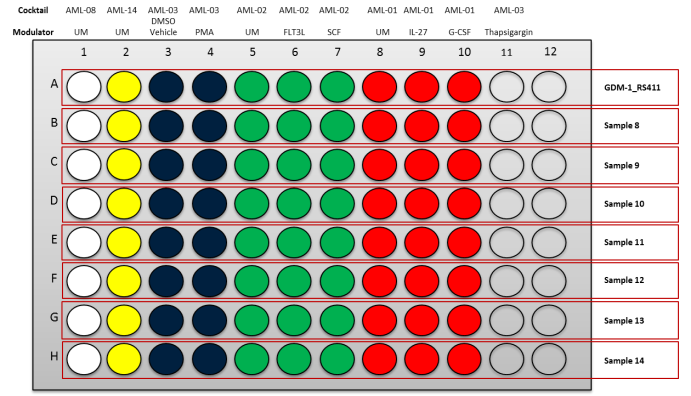 |
| 4-hour Apoptosis | 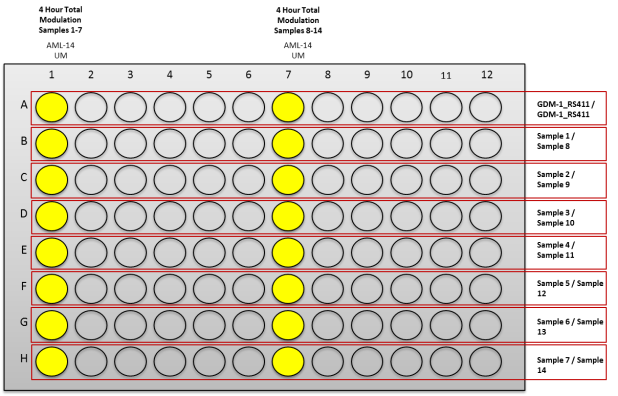 |
| 24-hour Apoptosis | 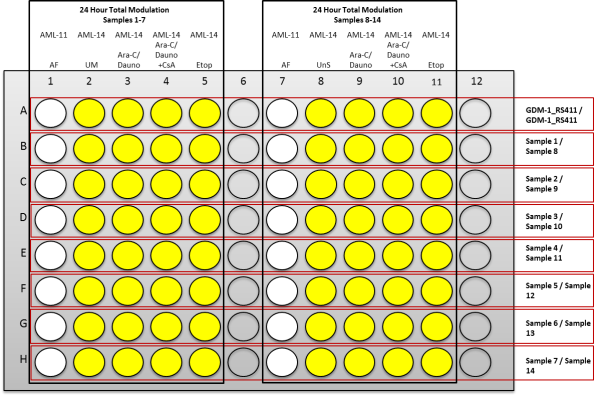 |

1. Quality Control Measures

Standard instrument controls (rainbow control particles, RCP) and cell line controls enabled the assessment of technical variability at the modulation, fixation, staining, and acquisition steps in the laboratory work flow thus allowing for the generation of reproducible results across operators, plates and time. These controls are essential in clinically applicable assays.

- - 1. Rainbow Control Particles (RCP)

Intra- and inter-cytometer variance and longitudinal consistency of instrument performance were monitored by including a single lot of 8-peak RCP beads on each plate across the entire experiment. These RCPs are commercially available from Spherotech (Lake Forest, IL). RCPs were plated on the last column of each plate. The data from these beads is used to both monitor the performance of the cytometers as well as to calibrate the fluorescence intensity values for data from the remaining wells on the plates (equivalent reference fluorochrome, ERF, calculation). Data from these wells was first gated to identify the 8 distinct intensity peaks. The median fluorescence intensity (MFI) value for each peak in each channel was computed. The coefficient of variation (CV) for each peak and channel combination was computed across all the plates.

The table below shows the CVs for all the three instruments used in the study when calculated across the experiment (%CV) and also within each plate (%CV by Plate) and also by all the plates collected on a given acquisition date (%CV by Day).

| **Channel** | **Peak** | **V96300490** | |  | **V96300493** | |  | **V96300766** | |  |
| --- | --- | --- | --- | --- | --- | --- | --- | --- | --- | --- |
|  |  | % CV | %CV By Plate | % CV By Day | % CV | %CV By Plate | % CV By Day | % CV | %CV By Plate | % CV By Day |
| FL1 | Peak1 | 7.52 | 4.63 | 4.78 | 8.81 | 4.37 | 4.65 | 7.33 | 4.44 | 4.69 |
|  | Peak2 | 1.73 | 0.96 | 1.04 | 1.57 | 0.95 | 1.01 | 2.35 | 1.12 | 1.21 |
|  | Peak3 | 2.13 | 0.99 | 1.13 | 1.53 | 0.85 | 0.93 | 2.32 | 1.18 | 1.24 |
|  | Peak4 | 2.09 | 0.96 | 1.10 | 1.50 | 0.82 | 0.89 | 2.28 | 1.19 | 1.25 |
|  | Peak5 | 2.04 | 0.95 | 1.11 | 1.51 | 0.79 | 0.87 | 2.18 | 1.17 | 1.22 |
|  | Peak6 | 2.02 | 0.94 | 1.10 | 1.54 | 0.82 | 0.91 | 2.18 | 1.18 | 1.23 |
|  | Peak7 | 2.00 | 0.95 | 1.10 | 1.51 | 0.82 | 0.90 | 2.14 | 1.18 | 1.23 |
|  | Peak8 | 2.01 | 0.95 | 1.10 | 1.44 | 0.80 | 0.88 | 1.81 | 1.12 | 1.17 |
| FL2 | Peak1 | 7.79 | 6.63 | 6.81 | 8.11 | 5.61 | 5.78 | 6.45 | 5.41 | 5.69 |
|  | Peak2 | 2.03 | 1.00 | 1.11 | 1.65 | 0.82 | 0.92 | 2.56 | 1.18 | 1.24 |
|  | Peak3 | 1.99 | 0.94 | 1.08 | 1.53 | 0.80 | 0.88 | 2.43 | 1.20 | 1.26 |
|  | Peak4 | 1.92 | 0.95 | 1.10 | 1.54 | 0.81 | 0.90 | 2.37 | 1.20 | 1.26 |
|  | Peak5 | 1.88 | 0.95 | 1.10 | 1.54 | 0.81 | 0.90 | 2.29 | 1.19 | 1.24 |
|  | Peak6 | 1.89 | 0.95 | 1.10 | 1.56 | 0.83 | 0.93 | 2.31 | 1.20 | 1.25 |
|  | Peak7 | 1.85 | 0.95 | 1.10 | 1.54 | 0.84 | 0.93 | 2.25 | 1.18 | 1.24 |
|  | Peak8 | 1.38 | 0.81 | 0.93 | 1.10 | 0.70 | 0.77 | 1.67 | 1.06 | 1.10 |
| FL3 | Peak1 | 8.53 | 6.87 | 7.24 | 10.48 | 6.71 | 6.97 | 8.12 | 6.53 | 6.94 |
|  | Peak2 | 2.04 | 1.04 | 1.19 | 1.78 | 0.99 | 1.07 | 2.70 | 1.20 | 1.27 |
|  | Peak3 | 1.97 | 0.96 | 1.10 | 1.51 | 0.82 | 0.92 | 2.60 | 1.20 | 1.25 |
|  | Peak4 | 1.94 | 0.95 | 1.10 | 1.50 | 0.81 | 0.90 | 2.55 | 1.20 | 1.26 |
|  | Peak5 | 1.88 | 0.94 | 1.10 | 1.50 | 0.80 | 0.89 | 2.47 | 1.20 | 1.25 |
|  | Peak6 | 1.86 | 0.94 | 1.09 | 1.53 | 0.83 | 0.93 | 2.46 | 1.20 | 1.25 |
|  | Peak7 | 1.78 | 0.93 | 1.08 | 1.51 | 0.83 | 0.92 | 2.37 | 1.18 | 1.23 |
|  | Peak8 | 1.28 | 0.78 | 0.91 | 1.09 | 0.69 | 0.77 | 1.73 | 1.05 | 1.09 |
| FL4 | Peak1 | 9.75 | 7.92 | 8.24 | 9.74 | 6.71 | 6.94 | 8.75 | 6.96 | 7.21 |
|  | Peak2 | 2.40 | 1.45 | 1.54 | 2.01 | 1.18 | 1.23 | 2.93 | 1.39 | 1.47 |
|  | Peak3 | 2.11 | 1.12 | 1.24 | 1.52 | 0.88 | 0.95 | 2.59 | 1.23 | 1.29 |
|  | Peak4 | 2.05 | 1.06 | 1.20 | 1.50 | 0.82 | 0.90 | 2.54 | 1.23 | 1.28 |
|  | Peak5 | 1.96 | 1.00 | 1.13 | 1.51 | 0.80 | 0.88 | 2.48 | 1.19 | 1.24 |
|  | Peak6 | 1.96 | 1.02 | 1.15 | 1.53 | 0.82 | 0.90 | 2.49 | 1.21 | 1.26 |
|  | Peak7 | 1.86 | 0.99 | 1.12 | 1.53 | 0.81 | 0.89 | 2.38 | 1.18 | 1.23 |
|  | Peak8 | 0.00 | 0.00 | 0.00 | 0.00 | 0.00 | 0.00 | 0.00 | 0.00 | 0.00 |
| FL5 | Peak1 | 1.39 | 0.97 | 1.02 | 1.61 | 0.90 | 0.95 | 2.09 | 1.24 | 1.32 |
|  | Peak2 | 1.44 | 0.89 | 0.97 | 1.73 | 0.75 | 0.80 | 2.23 | 1.21 | 1.31 |
|  | Peak3 | 1.51 | 0.91 | 1.00 | 1.71 | 0.79 | 0.85 | 2.14 | 1.23 | 1.33 |
|  | Peak4 | 1.53 | 0.92 | 1.01 | 1.73 | 0.80 | 0.87 | 2.16 | 1.27 | 1.36 |
|  | Peak5 | 1.53 | 0.91 | 1.00 | 1.73 | 0.79 | 0.86 | 2.09 | 1.24 | 1.34 |
|  | Peak6 | 1.55 | 0.92 | 1.01 | 1.75 | 0.82 | 0.89 | 2.16 | 1.26 | 1.36 |
|  | Peak7 | 1.50 | 0.90 | 0.99 | 1.71 | 0.82 | 0.88 | 2.10 | 1.24 | 1.33 |
|  | Peak8 | 0.00 | 0.00 | 0.00 | 0.00 | 0.00 | 0.00 | 0.00 | 0.00 | 0.00 |
| FL6 | Peak1 | 1.54 | 1.05 | 1.13 | 1.83 | 1.02 | 1.10 | 2.23 | 1.31 | 1.41 |
|  | Peak2 | 1.46 | 0.95 | 1.03 | 1.88 | 0.84 | 0.90 | 2.28 | 1.22 | 1.32 |
|  | Peak3 | 1.52 | 0.94 | 1.03 | 1.84 | 0.81 | 0.87 | 2.17 | 1.19 | 1.30 |
|  | Peak4 | 1.57 | 0.95 | 1.06 | 1.86 | 0.82 | 0.88 | 2.17 | 1.24 | 1.35 |
|  | Peak5 | 1.57 | 0.92 | 1.03 | 1.85 | 0.80 | 0.86 | 2.11 | 1.22 | 1.34 |
|  | Peak6 | 1.60 | 0.95 | 1.05 | 1.87 | 0.82 | 0.89 | 2.16 | 1.25 | 1.36 |
|  | Peak7 | 1.53 | 0.92 | 1.01 | 1.85 | 0.81 | 0.87 | 2.10 | 1.22 | 1.33 |
|  | Peak8 | 0.00 | 0.00 | 0.00 | 0.00 | 0.00 | 0.00 | 0.65 | 0.48 | 0.50 |
| FL7 | Peak1 | 1.42 | 1.01 | 1.15 | 1.62 | 1.20 | 1.31 | 1.81 | 1.37 | 1.45 |
|  | Peak2 | 1.34 | 0.92 | 1.02 | 1.37 | 0.81 | 0.87 | 1.95 | 1.21 | 1.27 |
|  | Peak3 | 1.46 | 0.98 | 1.08 | 1.34 | 0.81 | 0.89 | 2.00 | 1.30 | 1.37 |
|  | Peak4 | 1.50 | 1.00 | 1.10 | 1.35 | 0.80 | 0.89 | 2.01 | 1.29 | 1.36 |
|  | Peak5 | 1.44 | 0.94 | 1.04 | 1.36 | 0.79 | 0.88 | 2.00 | 1.26 | 1.34 |
|  | Peak6 | 1.46 | 0.95 | 1.05 | 1.36 | 0.79 | 0.88 | 2.00 | 1.26 | 1.34 |
|  | Peak7 | 1.45 | 0.88 | 1.00 | 1.40 | 0.77 | 0.85 | 2.03 | 1.23 | 1.34 |
|  | Peak8 | 0.00 | 0.00 | 0.00 | 0.00 | 0.00 | 0.00 | 0.36 | 0.28 | 0.29 |
| FL8 | Peak1 | 4.33 | 3.15 | 3.39 | 5.33 | 3.77 | 3.92 | 3.04 | 2.57 | 2.76 |
|  | Peak2 | 1.49 | 1.00 | 1.10 | 1.61 | 0.92 | 1.01 | 2.00 | 1.21 | 1.27 |
|  | Peak3 | 1.54 | 0.99 | 1.09 | 1.41 | 0.80 | 0.88 | 2.03 | 1.29 | 1.35 |
|  | Peak4 | 1.60 | 1.01 | 1.11 | 1.41 | 0.79 | 0.87 | 2.06 | 1.31 | 1.36 |
|  | Peak5 | 1.52 | 0.94 | 1.04 | 1.42 | 0.79 | 0.87 | 2.04 | 1.27 | 1.32 |
|  | Peak6 | 1.52 | 0.93 | 1.02 | 1.41 | 0.77 | 0.85 | 2.03 | 1.25 | 1.31 |
|  | Peak7 | 1.44 | 0.85 | 0.96 | 1.42 | 0.75 | 0.83 | 2.04 | 1.21 | 1.26 |
|  | Peak8 | 1.51 | 0.90 | 1.01 | 1.40 | 0.76 | 0.86 | 2.03 | 1.28 | 1.34 |

Additionally, all cytometers are qualified each day before use according to the manufacturer’s suggested quality control program as well as a more stringent internally developed quality control program documented in approved SOPs and performance specifications. Cytometers performing outside Nodality’s established performance specifications were taken off-line, corrective actions taken and documented and the instrument then verified prior to bringing back on-line for use.

- - 1. Cell Lines

Overall assay performance was monitored by running GDM1 and RS4;11 cell lines on every plate. Original cell lines were obtained from American Type Culture Collection (ATCC; Manassas, VA). A single batch of these cell lines were expanded in culture, cryopreserved, quality control tested and released following performance verification according to approved SOPs and appropriate release specifications.

The table below highlights the overall %CVs of modulated signaling, measured by U_u_ metric, e for the cell lines during the course of the entire experiment across all cytometers and all dates of acquisition.

| **Modulator** | **ModTime (Min)** | **Stain** | **Color** | % CV **for GDM-1** | % CV **for RS4;11** |
| --- | --- | --- | --- | --- | --- |
| AraC+Duano | 1440 | cPARP | Violet_B-A | 2.811 | 3.246 |
| AraC+Duano | 1440 | p-Chk2 | Red_C-A | 4.855 | 3.932 |
| Etoposide | 1440 | cPARP | Violet_B-A | 4.034 | 0.979 |
| Etoposide | 1440 | p-Chk2 | Red_C-A | 5.495 | 4.141 |
| FLT3L | 15 | p-Akt | Red_C-A | 4.210 | 2.728 |
| FLT3L | 15 | p-Erk | Blue_D-A | 7.256 | 6.669 |
| FLT3L | 15 | p-S6 | Blue_E-A | 3.113 | 3.398 |
| G-CSF | 15 | p-Stat1 | Blue_E-A | 4.805 | 7.176 |
| G-CSF | 15 | p-Stat3 | Blue_D-A | 5.990 | 5.071 |
| G-CSF | 15 | p-Stat5 | Red_C-A | 5.367 | 4.723 |
| IL-27 | 15 | p-Stat1 | Blue_E-A | 3.466 | 4.892 |
| IL-27 | 15 | p-Stat3 | Blue_D-A | 4.796 | 4.654 |
| IL-27 | 15 | p-Stat5 | Red_C-A | 5.779 | 5.036 |
| PMA | 15 | p-CREB | Blue_D-A | 1.473 | 2.788 |
| PMA | 15 | p-Erk | Red_C-A | 2.102 | 1.176 |
| PMA | 15 | p-S6 | Blue_E-A | 2.458 | 3.019 |
| SCF | 15 | p-Akt | Red_C-A | 4.716 | 8.642 |
| SCF | 15 | p-Erk | Blue_D-A | 14.036 | 18.699 |
| SCF | 15 | p-S6 | Blue_E-A | 3.909 | 8.400 |
| Thapsigargin | 15 | p-CREB | Blue_D-A | 4.018 | 4.845 |
| Thapsigargin | 15 | p-Erk | Red_C-A | 5.143 | 5.382 |
| Thapsigargin | 15 | p-S6 | Blue_E-A | 3.583 | 7.265 |

Using these two levels of controls (RCP for cytometer performance and cell lines control for assay performance) the majority (28/44) of the functional assay readout CVs were less than 5% and most of them (42/44) were less than 10% as expected across all days and batches for the study.

1. Flow Sample/Specimen Details
   1. Sample/Specimen Material Description

Refer to the manuscript for all details on the clinical samples used in this study. Pre-specified evaluability criteria are described in the Supplemental methods Section 1.1.

- 1. Sample Treatment(s) Description

Refer to the manuscript for details on the experiment.

Cryopreserved samples were processed in batches. Upon thaw, cells underwent a Ficoll-Hypaque gradient purification. The samples were plated into 96-well plates (75,000 cells/well) (see Figure below for an example plate layout) and then incubated with modulators, fixed, and permeabilized as previously described for the SCNP assay (Kornblau, et al. Clin Cancer Res 2010; Cesano, Spellmeyer Methods Mol Biol, 2014; Cesano, et al., Cytometry B, 2012). The samples were then incubated with a cocktail of fluorochrome-conjugated antibodies that recognize extracellular lineage markers and intracellular epitopes including phospho-epitopes within intracellular signaling molecules.

1. Data Analysis Details
   1. List-mode Data File

Single cell data were then acquired on one of three BD FACS CANTO II flow cytometer and the raw flow cytometry data files (called FCS files) were deposited on the Nodality File server for later analysis. The FCS files contain all events (including debris, cells, etc.) collected from the cytometer from each well acquired separated into individual files.

- 1. Compensation Details

Nodality flow cytometers are maintained through a daily QC program to monitor fluorescence, PMT voltages, and compensation allowing multiple instruments and platforms to be utilized if required (see reference 12 in manuscript for description of standardized “window of analysis”). For the experiments performed within this study, PMT voltages were set based upon a standard instrument setup QC procedure and compensation values for each pure dye reagent were established and monitored within this QC program.

All compensation is performed computationally after data acquisition.

- 1. Gating (Data Filtering) Details

The populations of interest are 1) “P1” which describes the leukemic cell population and 2) “Healthy P1” which describes the healthy cells within the P1 population.

- - 1. Gate Description

The gating definitions for P1 and Healthy P1 and CD34+ are as follows and as highlighted in the figure.

| 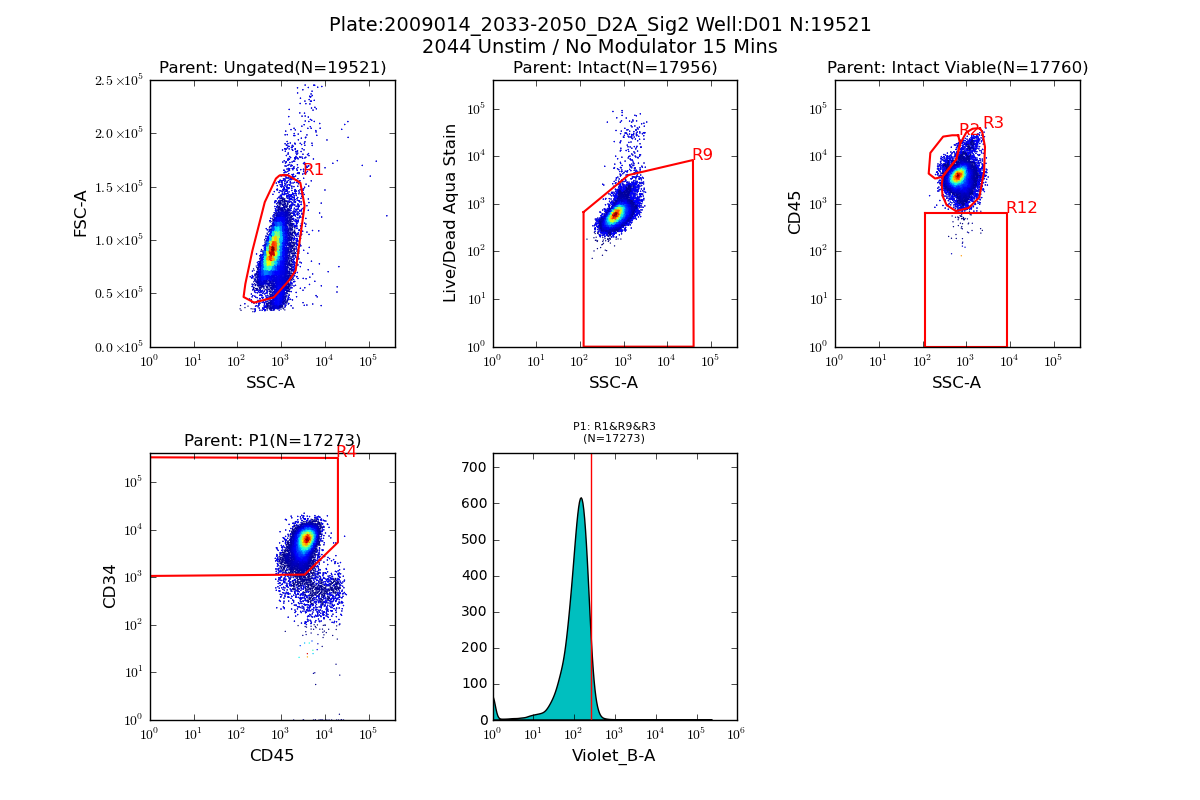 |
| --- |
| 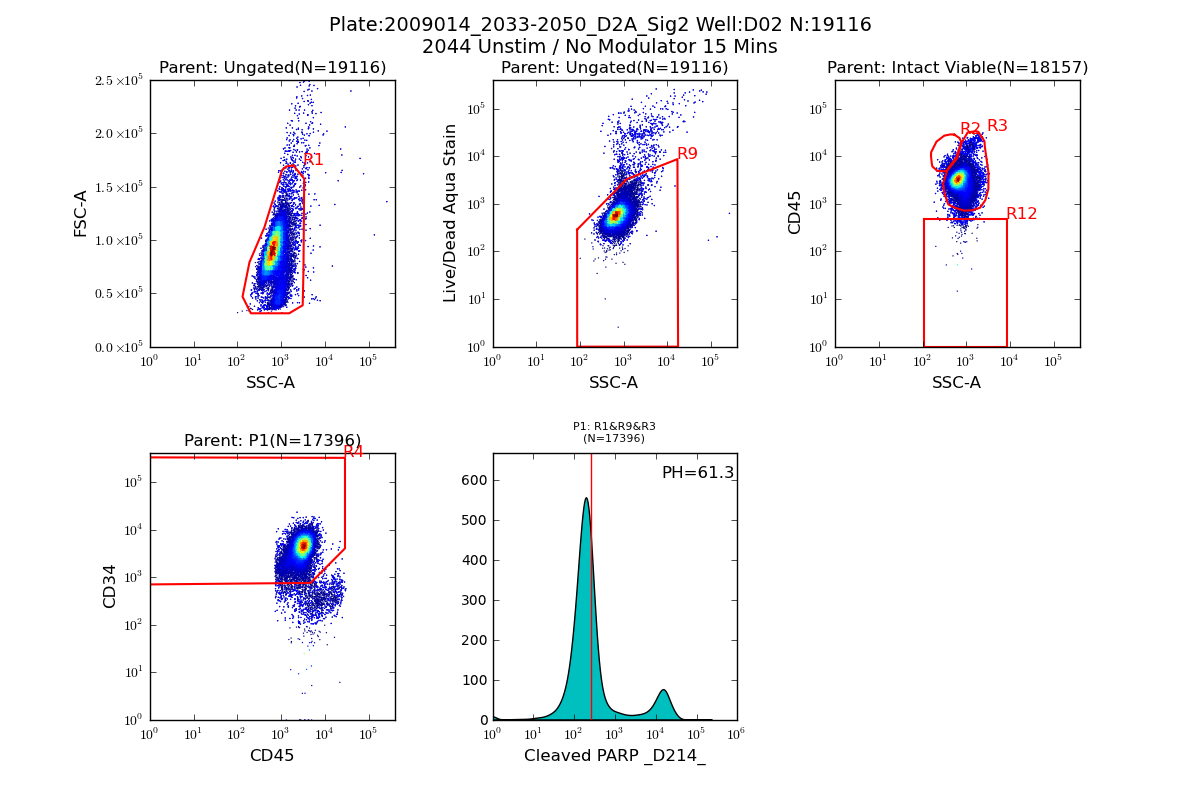 |

- - - 1. P1 gate

These are cells that are defined to be nucleated by light scatter (Region R1) but excluding all high SSC granulocytic forms (i.e., progranulocytes, metamyelocytes and myelocytes), are negative for amine aqua staining (Region R9), are CD45+ and express the SSC vs. CD45 characteristics of myeloid blasts and monocytoid cells (Region R3). The R3 gate will not include erythroid or progranulocytic cells. The Boolean equation used in WinList to derive these values is defined as: P1= (R1&R3&R9)

- - - 1. Healthy P1 gate

This gate captures the leukemic cells not undergoing apoptosis (cPARP negative or “healthy”). Each well contains an antibody against cPARP which will be used to determine the percentage of healthy cells in the P1 gate in each well. For each sample autofluorescence (AF) P1 values from the unmodulated 15’ timepoint wells will be used to compute a 98th percentile AF cutoff for that sample. cPARP staining in the other wells containing the same sample will use this cutoff to classify P1 events as cPARP positive (apoptotic P1) or cPARP negative (Healthy P1) cells.

- 1. Data Transformation Details and SCNP Metrics

Specific metrics were developed to describe and quantify the functional changes observed using the SCNP assay.

**Median fluorescence Intensity (MFI):**

MFI was computed from the fluorescence intensity levels of the cells.

**Equivalent Number of Reference Fluorophores (ERF) Metric:**

ERF a transformed value of the MFI values, was computed using a calibration line determined by fitting observations of a standardized set of 8-peak rainbow bead control particles for all fluorescent channels to standard values assigned by the manufacturer. ERF was used to standardize, qualify and monitor the instrument during setup, and to calibrate the raw fluorescence intensity readouts on a plate-by-plate basis and to control for instrument variability.

ERF values were then used to compute a variety of metrics to measure the biology of functional signaling proteins (see Supplemental Fig. S1). In the metric definitions that follow a = autofluorescence, u = unmodulated, and m = modulated.

**Log2Fold Change is defined as:**

$${log}_{2}Fold={log}_{2}\left[ \frac{{ERF}_{modulated}}{{ERF}_{unmodulated}} \right]$$

**Uu Metric:**

Computed as the Mann-Whitney U statistic comparing the intensity values for an antibody in the modulated and unmodulated wells that has been scaled to the unit interval (0,1) for a given cell population for a sample.

**Percent Healthy Metric:** $\mathbf{P}_{\mathbf{h}}^{\mathbf{Intact}}$

Percentage of leukemic blast cells that is negative for cPARP expression. The 98th percentile value for autofluorescence was used to determine the positive-negative split point.

| **Graphical Depiction of the process for calculation of the node-metrics.** |
| --- |
| 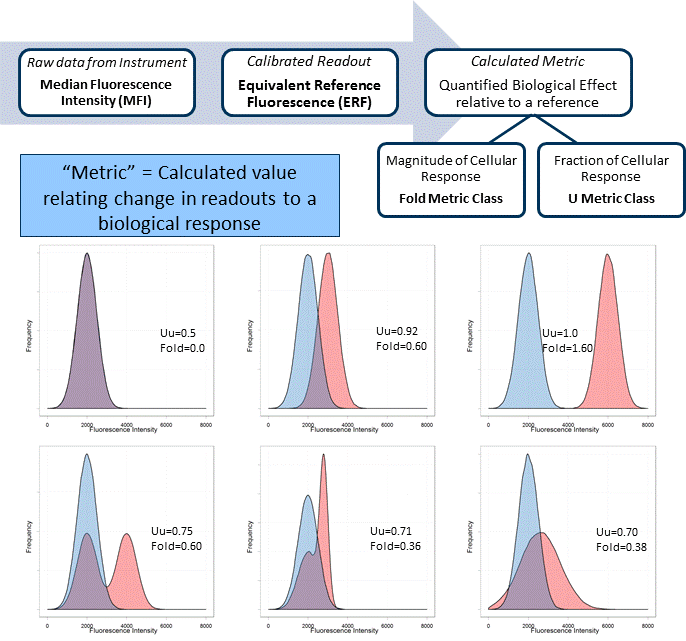 |

- - 1. Node-Metrics Used in the Training and Validation Studies

Calculated node-metrics for the nodes used in the final classifier have been provided as Supplemental Material with the publication.

FCS files, gating plots for the nodes used in the final classifier will be made available upon request.

- - 1. Gate Boundaries

Gating images showing the gating used for each well for the nodes used in the final classifier will be made available upon request.

1. Final sample disposition table

A table highlighting the final sample disposition of all samples processed as part of this study will be made available as a PDF upon request.

1. Relevant Experiment Information In Supplemental Materials

The following material can be found in the supplemental materials of the manuscript:

- Blinding and unblinding protocols
- Randomization for distribution of samples into training and validation sample sets
- Experimental details regarding selection of nodes included in the training phase.
- Baseline tables of the donors for the SWOG and ECOG data sets.
- Clinical data, variables, and endpoint definitions
- SWOG and ECOG treatment study details.
- Variable selection for training.
- Modeling methods.
- Verification of Candidate models using BM Verification Analysis Set.
- Imputation of clinical data for development of DX_CLINICAL2_
- Validation methodology
