# Supplementary material for: Cell Signaling-Based Classifier Predicts Response to Induction Therapy in Elderly Patients with Acute Myeloid Leukemia
Source: PLoS One. 2015 Apr 17;10(4):e0118485. doi: 10.1371/journal.pone.0118485 (PMC4401549; doi:10.1371/journal.pone.0118485)
Supplement: S1 Table — (DOCX) [file pone.0118485.s009.docx]

**S1 Table: SWOG and ECOG Treatment Study Details**

Clinical data, including baseline disease characteristics such as age, gender, cytogenetics, and AML onset (i.e., de-novo versus secondary AML), as well as induction and consolidation therapy information and associated outcomes, were collected and recorded for the parent SWOG studies S9031, S9333, S0112 and S0301 and ECOG studies E3993 and E3999 (Table A). The clinical data for the training, verification and validation sets were only released to Nodality after experimental data for the respective study phase had completed QC and data lock. Missing pretreatment data values were estimated by either pre-defined rules (blood counts) or nearest neighbor imputation for use in clinical predictors of response.

**Table A**

| **Study Number** | **Description of the Study** |  |
| --- | --- | --- |
| **SWOG-9031** | Short Title | Phase III placebo-controlled trial of Ara‑C/Dauno +/- G-CSF in elderly pts with untreated AML |
|  | Enrollment Dates | 1992-1994 |
|  | Number Enrolled | 234 |
|  | Number in Analysis Sets | 211 |
|  | Eligibility Criteria | Age 56+, newly diagnosed AML M1-7 (excluding M3), de novo or secondary |
|  | Treatment | **Induction:** Ara-C: 200 mg/m2/d CIV D1-7 and DNR: 45 mg/m2 IVP D1-3 plus: Either placebo or G‑CSF once daily **Post-CR:** Ara-C: 200 mg/m2/d CIV D1-5 DNR: 30 mg/m2 IVP D1-2 plus: Either placebo (Arm 1) or G-CSF once daily (Arm 2) |
|  | Outcomes | **CR Rates**: Placebo: 50%; G-CSF: 41% (p=0.89)/Overall: 45% (95/211) **RD Rate:** RD 35% **Fatal Ind Tox/Death w/in 7 days of treatment:** 20%/ not reported **Med RFS**: PBO: 9 mo; G-CSF: 8 mo (NS) **Med.** **OS**: Placebo: 9 mos; G-CSF: 6 mos. (p=.71) |
| **SWOG-9333** | Short Title | Phase III randomized trial of Mitoxantrone/Etoposide (ME) vs. Ara-C/Dauno (AD) in elderly pts with untreated AML |
|  | Enrollment Dates | 1995-1998 |
|  | Number Enrolled | 334 |
|  | Number in Analysis Sets | 328 included in analyses (Arm 1 [AD]: 162 enrolled, 161 included in analyses) |
|  | Eligibility Criteria | Age 56+, newly diagnosed AML M1-7 (excluding M3), de novo or secondary |
|  | Treatment | **Induction Patients from Arm 1 (only) eligible for SCNP study:** ***Arm 1-AD:*** Ara-C: 200 mg/m2/d CIV D1-7 and DNR: 45 mg/m2 IVP D1-3 plus: GM-CSF once daily ***Arm 2-ME:*** mitoxantrone 10 mg/m2/d x 5d and etoposide 100 mg/m2/d x 5d **Post-CR:** Ara-C: 200 mg/m2 CIV D1-5 and DNR: 30 mg/m2 IVP D1-2 plus: GM-CSF once daily |
|  | Outcomes | **CR Rates**: ME: 34%; AD: 43% (p=0.96) Overall: 38% (125/328) **RD Rate:** 39% **Fatal Ind Tox/ Death w/in 7 days of treatment:** 18%/ 7% **Med RFS**: ME: 7 mo; AD: 9 mo (NS) **OS @ 2 yrs**: ME: 11% ; AD: 19%. (p=.99) |
| **SWOG-S0112** | Short Title | Phase II trial of Ara‑C/Dauno in elderly pts with untreated AML (single arm) |
|  | Enrollment Dates | 2001-2003 |
|  | Number Enrolled | 71 |
|  | Number in Analysis Sets | 60 |
|  | Eligibility Criteria | Age 56+, newly diagnosed AML M1-7 (excluding M3), de novo or secondary |
|  | Treatment | **Induction:** Ara-C: 200 mg/m2/d CIV d1-7 and DNR: 45 mg/m2 IVP d1-3 plus: rhGM-GSF or G-CSF **Post-CR**: Ara-C: 200 mg/m2 CIV d1-5 and DNR: 45 mg/m2 IVP d1-2 |
|  | Outcomes | **CR Rate**: 38% (23/60) **RD Rate:** 45% **Fatal Ind Tox/ Death w/in 7 days of treatment:** 17%/7% **Med RFS**: 8 mo **Med**. **OS**: 7 mos. |
| SWOG-S0301 | Short Title | Phase II trial of Ara‑C/Dauno plus Cyclosporin-A in elderly pts with untreated AML (single arm) |
|  | Enrollment Dates | 2003-2006 |
|  | Number Enrolled | 55 |
|  | Number in Analysis Sets | 50 |
|  | Eligibility Criteria | Age 56+, newly diagnosed AML M1-7 (excluding M3), de novo or secondary |
|  | Treatment | **Induction**: Ara-C: 200 mg/m2/d CIV D1-7 and DNR: 45 mg/m2 IVP d1-3 plus: Cyclosporine 6mg/kg IV hrs -2 to 0, then 16mg/kg/d CIV d1-3; rhGM-GSF or G-CSF **Post-CR**: Ara-C: 200 mg/m2 CIV d1-5 and DNR: 45 mg/m2 IVP d1-2 plus: Cyclosporine 6mg/kg IV hrs -2 to 0 then 16mg/kg/d IV CIV d1-2 |
|  | Outcomes | **CR Rate**: 44% (22/50) **RD Rate:** 43% **Fatal Ind Tox/ Death w/in 7 days of treatment:** 12%/8% **Med RFS**: 14 mo **Med**. **OS**: 14 mos. |
| **ECOG-E3993** | Short Title | Comparing Three Standard Treatments in Older Adult Patients with Acute Non-Lymphocytic Leukemia and Studying the Effect of GM-CSF to See Whether It May Improve Initial Response to Chemotherapy (A Phase III Trial) |
|  | Enrollment Dates | 1993-1997 |
|  | Number Enrolled | 362 |
|  | Number in Analysis Sets | - |
|  | Eligibility Criteria | Age ≥56 M0-M7 AML No prior RT/CT De novo or secondary |
|  | Treatment | **Induction** (all pts): 1-2 cycles Ara-C (100 mg/m2/d) X 7 days PLUS-**RANDOMIZE:** Daunorubicin 45 mg/m2/d x 3d VS. Idarubicin 12 mg/m2/d x 3d VS. Mitoxantrone 12 mg/m2/d x 3 d. **Post-CR**: 1 cycle Ara-C (1.5 gm/m2) q. 12 hrs x 12 doses > 70 yrs. old: 6 doses |
|  | Outcomes | "Comparison of CR rates among 3 induction regimens (primary endpoint): no statistically significant difference. Comparison of CR rates btwn GM-CSF vs. PB0 (co-primary endpoint): no statistically significant difference.” |
| **ECOG-E3999** | Short Title | Daunorubicin & Cytarabine +/- Zosuquidar in Treating Older Patients With Newly Diagnosed Acute Myeloid Leukemia or Refractory Anemia |
|  | Enrollment Dates | 2002-2006 |
|  | Number Enrolled | 449 |
|  | Number in Analysis Sets | - |
|  | Eligibility Criteria | Age 60+ AML M0-M7, exclude M3 No prior CT De novo or secondary |
|  | Treatment | **Induction** (all pts): 1-2 cycles 7+3 Ara-C (100 mg/m2/d)/Daunorubicin (45 mg/m2/d) **RANDOMIZE:** zosuquidar trihydrochloride IV days 1-3 vs. PBO Growth factor support: G-CSF or GM-CSF start D12 to ANC recovery **Consolidation I** (all pts in CR): 1 cycle Ara-C (1.5 gm/m2) q. 12 hrs x 12 doses Growth factor support: G-CSF or GM-CSF start D7 to ANC recovery **Consolidation II** (all pts in CR): repeat induction regimen x 1 cycle |
|  | Outcomes | No difference in median OS (primary endpoint). No difference in median O.S. of pts with high P-gp status. |
