# Supplementary material for: Cell Signaling-Based Classifier Predicts Response to Induction Therapy in Elderly Patients with Acute Myeloid Leukemia
Source: PLoS One. 2015 Apr 17;10(4):e0118485. doi: 10.1371/journal.pone.0118485 (PMC4401549; doi:10.1371/journal.pone.0118485)
Supplement: S2 Table — (DOCX) [file pone.0118485.s010.docx]

**S2 Table: Baseline characteristics of patients on SWOG trials: SCNP‑evaluable vs. all other eligible, evaluable who did not decline consent for specimen.**

| **Patient/Disease Characteristics** | **Sub-Groups** | **SCNP-evaluable (N=213)** | | **All Others^a^ (N=294)** | | **P** | **Test** |
| --- | --- | --- | --- | --- | --- | --- | --- |
|  |  | **N** | **%** | **N** | **%** |  |  |
| Study/Arm | S9031/AD | 45 | 21.1% | 68 | 23.1% | <.0001 | ChiSq |
|  | S9031/AD+G | 27 | 12.7% | 84 | 28.6% |  |  |
|  | S9333/AD | 70 | 32.9% | 91 | 31.0% |  |  |
|  | S0112/AD | 29 | 13.6% | 30 | 10.2% |  |  |
|  | S0301/AD+C | 42 | 19.7% | 21 | 7.1% |  |  |
| Sex | F | 94 | 44.1% | 131 | 44.6% | 0.93 | Fisher |
|  | M | 119 | 55.9% | 163 | 55.4% |  |  |
| AML Onset | De Novo | 162 | 76.1% | 227 | 77.5% | 0.75 | Fisher |
|  | Secondary | 51 | 23.9% | 66 | 22.5% |  |  |
|  | Unknown | 0 | . | 1 | . |  |  |
| SWOG PS | 0 | 58 | 27.5% | 69 | 24.0% | 0.41 | ChiSq |
|  | 1 | 98 | 46.4% | 148 | 51.6% |  |  |
|  | 2 | 31 | 14.7% | 47 | 16.4% |  |  |
|  | 3 | 24 | 11.4% | 23 | 8.0% |  |  |
|  | Unknown | 2 | . | 7 | . |  |  |
| Cytogenetics | Normal | 73 | 42.7% | 75 | 34.7% | 0.0037 | ChiSq |
|  | Nml+Nonclonal | 4 | 2.3% | 9 | 4.2% |  |  |
|  | del5q7q | 24 | 14.0% | 61 | 28.2% |  |  |
|  | CBF | 16 | 9.4% | 9 | 4.2% |  |  |
|  | Other | 54 | 31.6% | 62 | 28.7% |  |  |
|  | Unknown | 42 | . | 78 | . |  |  |
| Age (Years) | (Min, Max), Median | (57, 69), 68.6 |  | (56, 85), 67.1 |  | 0.13 | Wilcoxon |
| BM blasts (%) | (Min, Max), Median | (6, 99), 68 |  | (0, 99), 60 |  | 0.003 | Wilcoxon |
| WBC (10^9^/L) | (Min, Max), Median | (0.7, 298), 22 |  | (0.6, 294), 5.1 |  | <.0001 | Wilcoxon |
| PB blasts (%) | (Min, Max), Median | (0,99), 40 |  | (0, 95), 13 |  | <.0001 | Wilcoxon |

a Includes N=241 with insufficient material for assay and N=53 assayed but with nonevaluable results (see Figure 1)
