# Supplementary material for: Cell Signaling-Based Classifier Predicts Response to Induction Therapy in Elderly Patients with Acute Myeloid Leukemia
Source: PLoS One. 2015 Apr 17;10(4):e0118485. doi: 10.1371/journal.pone.0118485 (PMC4401549; doi:10.1371/journal.pone.0118485)
Supplement: S3 Table — (DOCX) [file pone.0118485.s011.docx]

**S3 Table: SCNP node-metrics that are used as inputs to DX_SCNP_ for the BM Training, PB Training, BM Validation and PB Validation Analysis Sets along with the response information, tissue type and analysis set.**

| Patient ID | Sample | AraC+Dauno (24 Hours) → CD34 \| Uu | AraC+Dauno (24 Hours) → cPARP \| Uu | Response | Set | Tissue |
| --- | --- | --- | --- | --- | --- | --- |
| Train063 | 2004 | 0.3584 | 0.5 | CR/CRi | Training | BM |
| Train027 | 2021 | 0.3871 | 0.5729 | CR/CRi | Training | BM |
| Train074 | 2027 | 0.4469 | 0.5522 | CR/CRi | Training | BM |
| Train057 | 2041 | 0.3305 | 0.525 | CR/CRi | Training | BM |
| Train039 | 2043 | 0.3216 | 0.6984 | RD | Training | BM |
| Train037 | 2044 | 0.4553 | 0.534 | RD | Training | BM |
| Train072 | 2048 | 0.4884 | 0.5164 | RD | Training | BM |
| Train077 | 2075 | 0.2449 | 0.7132 | CR/CRi | Training | BM |
| Train018 | 2108 | 0.2694 | 0.5548 | CR/CRi | Training | BM |
| Train091 | 2112 | 0.2866 | 0.585 | CR/CRi | Training | BM |
| Train080 | 2113 | 0.3421 | 0.5803 | CR/CRi | Training | BM |
| Train044 | 2121 | 0.4841 | 0.524 | RD | Training | BM |
| Train003 | 2129 | 0.2639 | 0.5527 | CR/CRi | Training | BM |
| Train056 | 2151 | 0.4662 | 0.5784 | CR/CRi | Training | BM |
| Train049 | 2158 | 0.3819 | 0.5 | CR/CRi | Training | BM |
| Train065 | 2179 | 0.387 | 0.5 | RD | Training | BM |
| Train002 | 2220 | 0.2533 | 0.6604 | CR/CRi | Training | BM |
| Train009 | 2253 | 0.2751 | 0.6112 | RD | Training | BM |
| Train103 | 2255 | 0.1877 | 0.5774 | CR/CRi | Training | BM |
| Train014 | 2257 | 0.1625 | 0.5419 | CR/CRi | Training | BM |
| Train045 | 2266 | 0.3843 | 0.6435 | CR/CRi | Training | BM |
| Train075 | 2273 | 0.5 | 0.5297 | RD | Training | BM |
| Train006 | 2291 | 0.4482 | 0.5672 | CR/CRi | Training | BM |
| Train108 | 2295 | 0.1858 | 0.7287 | CR/CRi | Training | BM |
| Train048 | 2296 | 0.3512 | 0.7469 | CR/CRi | Training | BM |
| Train032 | 2297 | 0.3629 | 0.6483 | RD | Training | BM |
| Train070 | 2302 | 0.1975 | 0.5 | CR/CRi | Training | BM |
| Train031 | 2306 | 0.0962 | 0.5478 | CR/CRi | Training | BM |
| Train099 | 2315 | 0.2756 | 0.7284 | CR/CRi | Training | BM |
| Train043 | 2332 | 0.42 | 0.5349 | CR/CRi | Training | BM |
| Train050 | 2338 | 0.4448 | 0.5291 | RD | Training | BM |
| Train058 | 2341 | 0.4319 | 0.577 | CR/CRi | Training | BM |
| Train013 | 2352 | 0.2835 | 0.7707 | CR/CRi | Training | BM |
| Train055 | 2370 | 0.2331 | 0.5 | CR/CRi | Training | BM |
| Train030 | 2372 | 0.414 | 0.5619 | RD | Training | BM |
| Train034 | 2389 | 0.3809 | 0.5583 | RD | Training | BM |
| Train017 | 2395 | 0.1635 | 0.5395 | CR/CRi | Training | BM |
| Train024 | 2398 | 0.4787 | 0.5 | RD | Training | BM |
| Train012 | 2402 | 0.3077 | 0.5997 | CR/CRi | Training | BM |
| Train101 | 2408 | 0.1555 | 0.627 | CR/CRi | Training | BM |
| Train028 | 2421 | 0.2373 | 0.5627 | CR/CRi | Training | BM |
| Train062 | 2429 | 0.2084 | 0.5931 | CR/CRi | Training | BM |
| Train100 | 2433 | 0.2266 | 0.5895 | CR/CRi | Training | BM |
| Train030 | 2007 | 0.463 | 0.5774 | RD | Training | PB |
| Train012 | 2028 | 0.2827 | 0.5743 | CR/CRi | Training | PB |
| Train089 | 2031 | 0.3904 | 0.6575 | CR/CRi | Training | PB |
| Train103 | 2032 | 0.1992 | 0.5794 | CR/CRi | Training | PB |
| Train061 | 2061 | 0.3723 | 0.5886 | RD | Training | PB |
| Train085 | 2066 | 0.2336 | 0.7488 | CR/CRi | Training | PB |
| Train088 | 2077 | 0.3422 | 0.7695 | CR/CRi | Training | PB |
| Train079 | 2078 | 0.2094 | 0.5 | RD | Training | PB |
| Train037 | 2085 | 0.4395 | 0.5561 | RD | Training | PB |
| Train074 | 2094 | 0.4474 | 0.5 | CR/CRi | Training | PB |
| Train045 | 2111 | 0.3656 | 0.6191 | CR/CRi | Training | PB |
| Train043 | 2120 | 0.442 | 0.5721 | CR/CRi | Training | PB |
| Train052 | 2126 | 0.3691 | 0.6417 | CR/CRi | Training | PB |
| Train017 | 2154 | 0.2278 | 0.5608 | CR/CRi | Training | PB |
| Train065 | 2159 | 0.3671 | 0.5733 | RD | Training | PB |
| Train066 | 2160 | 0.2556 | 0.7211 | CR/CRi | Training | PB |
| Train018 | 2167 | 0.2668 | 0.5404 | CR/CRi | Training | PB |
| Train034 | 2168 | 0.375 | 0.5818 | RD | Training | PB |
| Train044 | 2189 | 0.4933 | 0.5293 | RD | Training | PB |
| Train002 | 2198 | 0.2901 | 0.7347 | CR/CRi | Training | PB |
| Train073 | 2199 | 0.239 | 0.6261 | CR/CRi | Training | PB |
| Train096 | 2214 | 0.3876 | 0.5346 | RD | Training | PB |
| Train015 | 2225 | 0.3643 | 0.6917 | CR/CRi | Training | PB |
| Train028 | 2229 | 0.2699 | 0.5651 | CR/CRi | Training | PB |
| Train060 | 2237 | 0.2803 | 0.6428 | CR/CRi | Training | PB |
| Train046 | 2243 | 0.354 | 0.6155 | RD | Training | PB |
| Train069 | 2247 | 0.2341 | 0.5767 | RD | Training | PB |
| Train091 | 2250 | 0.201 | 0.6036 | CR/CRi | Training | PB |
| Train036 | 2251 | 0.2679 | 0.723 | CR/CRi | Training | PB |
| Train021 | 2271 | 0.1904 | 0.6088 | CR/CRi | Training | PB |
| Train025 | 2272 | 0.3232 | 0.7678 | CR/CRi | Training | PB |
| Train095 | 2281 | 0.2205 | 0.5 | CR/CRi | Training | PB |
| Train107 | 2287 | 0.1687 | 0.6125 | CR/CRi | Training | PB |
| Train048 | 2288 | 0.4586 | 0.8373 | CR/CRi | Training | PB |
| Train054 | 2313 | 0.2893 | 0.6159 | CR/CRi | Training | PB |
| Train039 | 2316 | 0.4026 | 0.5611 | RD | Training | PB |
| Train029 | 2319 | 0.2573 | 0.5793 | CR/CRi | Training | PB |
| Train086 | 2320 | 0.1875 | 0.529 | CR/CRi | Training | PB |
| Train003 | 2328 | 0.176 | 0.5864 | CR/CRi | Training | PB |
| Train092 | 2336 | 0.3631 | 0.5713 | RD | Training | PB |
| Train106 | 2343 | 0.2421 | 0.5 | CR/CRi | Training | PB |
| Train100 | 2350 | 0.2636 | 0.6528 | CR/CRi | Training | PB |
| Train059 | 2353 | 0.1693 | 0.5614 | CR/CRi | Training | PB |
| Train077 | 2358 | 0.2381 | 0.7286 | CR/CRi | Training | PB |
| Train099 | 2362 | 0.3188 | 0.7348 | CR/CRi | Training | PB |
| Train075 | 2364 | 0.4784 | 0.5111 | RD | Training | PB |
| Train004 | 2367 | 0.3275 | 0.5489 | CR/CRi | Training | PB |
| Train005 | 2397 | 0.3934 | 0.6617 | CR/CRi | Training | PB |
| Train001 | 2401 | 0.0933 | 0.7094 | CR/CRi | Training | PB |
| Train076 | 2403 | 0.0844 | 0.6558 | CR/CRi | Training | PB |
| Train084 | 2405 | 0.2024 | 0.6828 | CR/CRi | Training | PB |
| Train020 | 2409 | 0.4133 | 0.5117 | CR/CRi | Training | PB |
| Train011 | 2430 | 0.235 | 0.6275 | CR/CRi | Training | PB |
| Train072 | 2438 | 0.4769 | 0.564 | RD | Training | PB |
| Train027 | 2440 | 0.3462 | 0.6174 | CR/CRi | Training | PB |
| Train108 | 2450 | 0.1605 | 0.6474 | CR/CRi | Training | PB |
| Train101 | 2465 | 0.1911 | 0.6312 | CR/CRi | Training | PB |
| Valid006 | 2019 | 0.443 | 0.5937 | RD | Validation | BM |
| Valid014 | 2020 | 0.3819 | 0.5083 | RD | Validation | BM |
| Valid028 | 2050 | 0.3367 | 0.7264 | CR/CRi | Validation | BM |
| Valid018 | 2086 | 0.2781 | 0.5227 | RD | Validation | BM |
| Valid044 | 2092 | 0.4095 | 0.5991 | CR/CRi | Validation | BM |
| Valid079 | 2107 | 0.4096 | 0.6152 | CR/CRi | Validation | BM |
| Valid091 | 2125 | 0.2654 | 0.6726 | CR/CRi | Validation | BM |
| Valid041 | 2130 | 0.0922 | 0.6954 | CR/CRi | Validation | BM |
| Valid011 | 2156 | 0.2116 | 0.5237 | CR/CRi | Validation | BM |
| Valid096 | 2166 | 0.4343 | 0.5733 | CR/CRi | Validation | BM |
| Valid074 | 2173 | 0.3175 | 0.5652 | CR/CRi | Validation | BM |
| Valid038 | 2183 | 0.2027 | 0.6143 | CR/CRi | Validation | BM |
| Valid072 | 2185 | 0.4102 | 0.5527 | CR/CRi | Validation | BM |
| Valid016 | 2223 | 0.2809 | 0.7343 | CR/CRi | Validation | BM |
| Valid052 | 2232 | 0.3036 | 0.5595 | CR/CRi | Validation | BM |
| Valid068 | 2239 | 0.3251 | 0.6913 | CR/CRi | Validation | BM |
| Valid002 | 2245 | 0.4376 | 0.5262 | CR/CRi | Validation | BM |
| Valid065 | 2259 | 0.2569 | 0.5882 | CR/CRi | Validation | BM |
| Valid064 | 2262 | 0.2638 | 0.7258 | CR/CRi | Validation | BM |
| Valid077 | 2268 | 0.4282 | 0.522 | CR/CRi | Validation | BM |
| Valid060 | 2270 | 0.4299 | 0.6019 | RD | Validation | BM |
| Valid059 | 2274 | 0.3286 | 0.6568 | CR/CRi | Validation | BM |
| Valid043 | 2280 | 0.3837 | 0.5123 | RD | Validation | BM |
| Valid037 | 2314 | 0.1769 | 0.5768 | CR/CRi | Validation | BM |
| Valid067 | 2324 | 0.4404 | 0.5751 | RD | Validation | BM |
| Valid103 | 2334 | 0.2984 | 0.5054 | CR/CRi | Validation | BM |
| Valid022 | 2335 | 0.3732 | 0.5333 | CR/CRi | Validation | BM |
| Valid105 | 2345 | 0.3426 | 0.7285 | CR/CRi | Validation | BM |
| Valid100 | 2346 | 0.2478 | 0.5844 | CR/CRi | Validation | BM |
| Valid099 | 2355 | 0.3006 | 0.723 | RD | Validation | BM |
| Valid019 | 2365 | 0.242 | 0.5678 | CR/CRi | Validation | BM |
| Valid005 | 2369 | 0.3539 | 0.5103 | CR/CRi | Validation | BM |
| Valid012 | 2371 | 0.3835 | 0.5474 | RD | Validation | BM |
| Valid047 | 2375 | 0.396 | 0.5673 | RD | Validation | BM |
| Valid061 | 2390 | 0.439 | 0.6215 | CR/CRi | Validation | BM |
| Valid055 | 2417 | 0.3571 | 0.5704 | CR/CRi | Validation | BM |
| Valid075 | 2436 | 0.2666 | 0.5534 | CR/CRi | Validation | BM |
| Valid057 | 2445 | 0.494 | 0.6504 | CR/CRi | Validation | BM |
| Valid095 | 2451 | 0.2453 | 0.65 | CR/CRi | Validation | BM |
| Valid104 | 2456 | 0.4589 | 0.5847 | CR/CRi | Validation | BM |
| Valid004 | 2458 | 0.2937 | 0.6751 | CR/CRi | Validation | BM |
| Valid085 | 2468 | 0.3563 | 0.603 | RD | Validation | BM |
| Valid043 | 2003 | 0.3002 | 0.5179 | RD | Validation | PB |
| Valid091 | 2005 | 0.2449 | 0.6101 | CR/CRi | Validation | PB |
| Valid038 | 2008 | 0.166 | 0.5883 | CR/CRi | Validation | PB |
| Valid078 | 2010 | 0.3351 | 0.5859 | CR/CRi | Validation | PB |
| Valid105 | 2012 | 0.3186 | 0.8178 | CR/CRi | Validation | PB |
| Valid083 | 2018 | 0.19 | 0.7275 | CR/CRi | Validation | PB |
| Valid103 | 2026 | 0.2588 | 0.5643 | CR/CRi | Validation | PB |
| Valid085 | 2033 | 0.1647 | 0.7138 | RD | Validation | PB |
| Valid052 | 2035 | 0.2975 | 0.5347 | CR/CRi | Validation | PB |
| Valid053 | 2055 | 0.2318 | 0.641 | CR/CRi | Validation | PB |
| Valid005 | 2071 | 0.2313 | 0.598 | CR/CRi | Validation | PB |
| Valid017 | 2072 | 0.3718 | 0.5294 | CR/CRi | Validation | PB |
| Valid100 | 2076 | 0.1829 | 0.5516 | CR/CRi | Validation | PB |
| Valid021 | 2095 | 0.3597 | 0.6498 | CR/CRi | Validation | PB |
| Valid097 | 2106 | 0.2907 | 0.6528 | CR/CRi | Validation | PB |
| Valid027 | 2110 | 0.4311 | 0.496 | CR/CRi | Validation | PB |
| Valid004 | 2118 | 0.3641 | 0.5267 | CR/CRi | Validation | PB |
| Valid079 | 2127 | 0.3789 | 0.6551 | CR/CRi | Validation | PB |
| Valid068 | 2133 | 0.1896 | 0.7814 | CR/CRi | Validation | PB |
| Valid066 | 2138 | 0.3451 | 0.4578 | CR/CRi | Validation | PB |
| Valid075 | 2144 | 0.2268 | 0.5412 | CR/CRi | Validation | PB |
| Valid006 | 2146 | 0.3852 | 0.5711 | RD | Validation | PB |
| Valid025 | 2148 | 0.3473 | 0.6323 | RD | Validation | PB |
| Valid104 | 2161 | 0.4367 | 0.5416 | CR/CRi | Validation | PB |
| Valid011 | 2162 | 0.1732 | 0.47 | CR/CRi | Validation | PB |
| Valid024 | 2211 | 0.2123 | 0.6757 | CR/CRi | Validation | PB |
| Valid002 | 2215 | 0.4494 | 0.5171 | CR/CRi | Validation | PB |
| Valid051 | 2228 | 0.1589 | 0.6185 | CR/CRi | Validation | PB |
| Valid090 | 2241 | 0.2863 | 0.7541 | CR/CRi | Validation | PB |
| Valid037 | 2252 | 0.2557 | 0.5825 | CR/CRi | Validation | PB |
| Valid048 | 2254 | 0.4231 | 0.6071 | CR/CRi | Validation | PB |
| Valid016 | 2260 | 0.2534 | 0.682 | CR/CRi | Validation | PB |
| Valid081 | 2263 | 0.147 | 0.5422 | RD | Validation | PB |
| Valid020 | 2301 | 0.4719 | 0.5943 | CR/CRi | Validation | PB |
| Valid063 | 2323 | 0.1645 | 0.6196 | CR/CRi | Validation | PB |
| Valid034 | 2327 | 0.4049 | 0.6079 | CR/CRi | Validation | PB |
| Valid069 | 2331 | 0.5397 | 0.4809 | RD | Validation | PB |
| Valid062 | 2337 | 0.102 | 0.5543 | CR/CRi | Validation | PB |
| Valid013 | 2339 | 0.2891 | 0.6239 | CR/CRi | Validation | PB |
| Valid010 | 2342 | 0.2766 | 0.6008 | CR/CRi | Validation | PB |
| Valid059 | 2363 | 0.3279 | 0.7122 | CR/CRi | Validation | PB |
| Valid018 | 2383 | 0.3423 | 0.4994 | RD | Validation | PB |
| Valid054 | 2400 | 0.3492 | 0.6442 | CR/CRi | Validation | PB |
| Valid072 | 2414 | 0.4035 | 0.5385 | CR/CRi | Validation | PB |
| Valid082 | 2425 | 0.2059 | 0.5824 | RD | Validation | PB |
| Valid032 | 2427 | 0.3397 | 0.7973 | RD | Validation | PB |
| Valid050 | 2434 | 0.4674 | 0.5862 | CR/CRi | Validation | PB |
| Valid047 | 2448 | 0.4659 | 0.6197 | RD | Validation | PB |
| Valid023 | 2452 | 0.3091 | 0.5181 | CR/CRi | Validation | PB |
| Valid056 | 2453 | 0.4577 | 0.5756 | CR/CRi | Validation | PB |
| Valid058 | 2460 | 0.3604 | 0.6285 | CR/CRi | Validation | PB |
| Valid102 | 2467 | 0.3836 | 0.6289 | CR/CRi | Validation | PB |
| Valid019 | 2469 | 0.2264 | 0.6034 | CR/CRi | Validation | PB |
